# Hybrid Hairy Two-Dimensional Nanostructures with Tunable Morphologies by Inclusion Crystallization of Lead Bromide Complexes with Polystyrene-*block*-poly(ethylene oxide)

**DOI:** 10.1021/acs.langmuir.5c03419

**Published:** 2025-11-18

**Authors:** Ya-Sen Sun, Bo-Cheng Zhao, Chun-Chuen Yang, Orion Shih, Chun-Yu Chen, Chun-Jen Su, Jhih-Min Lin

**Affiliations:** † Department of Chemical Engineering, 34911 National Cheng Kung University, Tainan 701, Taiwan; ‡ Department of Physics, National Central University, Taoyuan 320317, Taiwan; § National Synchrotron Radiation Research Center, Hsinchu 30076, Taiwan

## Abstract

We report the formation of hybrid hairy nanostructures composed of polystyrene-*block*-poly­(ethylene oxide) (PS-*b*-PEO) and lead bromide (PbBr_2_) complexes in 1,3,5-trimethylbenzene (TMB). In this system, TMB acts as a selective solvent (good for the PS block and bad for the PEO block) while serving as a nonsolvent for PbBr_2_. Although PbBr_2_ is insoluble in neat TMB, its complexation with PS-*b*-PEO promotes the formation of the [PbBr_3_]^−^ and [PbBr_4_]^2–^ complexes. These complexes coordinate with the ether groups of the PEO block, forming host–guest interactions that drive the inclusion crystallization of two-dimensional (2D) hybrid nanostructures, including irregular nanosheets and polygonal nanoplates. The morphology of these nanostructures strongly depends on the PS-*b*-PEO/PbBr_2_ weight ratio. Higher ratios (20/100 and 20/200) favor irregular nanosheets, while lower ratios (20/10) favor polygonal nanoplates. Structural analysis reveals that the irregular nanosheets adopt an orthorhombic *Cmca* lattice (*a* = 23.53 Å, *b* = 4.20 Å, *c* = 33.22 Å), whereas the polygonal nanoplates exhibit a hexagonal *P*6*mm* lattice (*a*_hex_ = 13.954 Å, γ = 120°). Both types of 2D structures are decorated with ultrasmall PbBr_2_ nanoparticles encapsulated by PS-*b*-PEO chains. The PEO blocks coordinate to nanoparticle surfaces, while PS blocks swell in the TMB. This synergistic process integrates PbBr_2_ complexation, host–guest coordination, inclusion crystallization, and block copolymer-mediated nanoparticle assembly. In spin-coated films, polygonal nanoplates preferentially adopt edge-on orientations, while irregular nanosheets lie flat-on. These findings offer insights into block copolymer templating of 2D organic–inorganic hybrid nanostructures.

## Graphical abstract

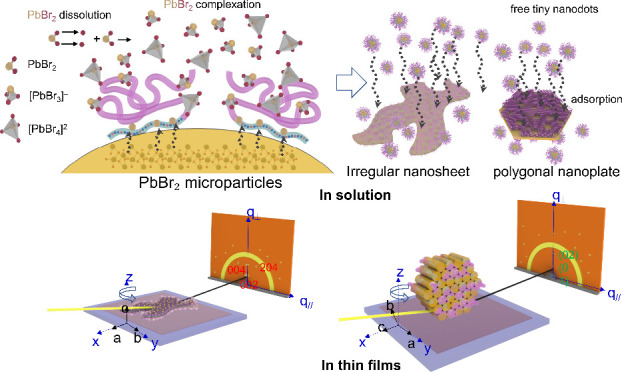

## Introduction

Due to their morphological diversity, controllable order, and tunable nanodomains,^1^ the self-assembly of block copolymers (BCPs) in solution has provided a versatile platform for tailoring the structures and morphologies of inorganic nanomaterials, such as carbon, metal, metal oxide, and semiconductors.^2^ In particular, solvent selectivity, polymer concentrations, and the addition of additives (such as ions or polymers) have been found to effectively modulate diverse morphologies.^3,4^ These morphologies can be either thermodynamically stable or kinetically trapped metastable structures.^5–9^ Although the formation of kinetically trapped metastable structures is complex and challenging to control, it is typically path-dependent and strongly influenced by synthesis processes. However, if the kinetic pathways leading to micelles with diverse morphologies can be well understood, accounting for nonergodicity,^10^ then precise kinetic control over the formation of metastable phases could become highly advantageous in practical applications.^8–10^

Furthermore, if one of the constituent blocks in a block copolymer is crystallizable, unique micellar structures, such as unidirectional nanoribbons and nanowires or bidirectional platelets, can form through crystallization-driven self-assembly (CDSA).^11–21^ Another advantage of incorporating a crystallizable block in a block copolymer is the ability to facilitate inclusion crystallization of poly­(ethylene oxide) chains with ionic complexes.^22–25^ Through this mechanism, one-dimensional (1D) nanoribbons and two-dimensional (2D) platelets have been successfully obtained via the inclusion crystallization of ionic complexes and polymers. These anisotropic nanostructures contrast significantly with the conventional morphologies of block copolymer self-assembly in solution, which typically yield spherical micelles, cylindrical micelles, bicontinuous cubosomes, or vesicles.^1,3,4^ On a broader scale, ultrathin nanocrystals have also been shown to form complex multidimensional superstructures through noncovalent interparticle interactions and self-assembly kinetics, which can be regulated by solvent quality, ligand type, and the nucleation and growth processes to yield diverse crystalline morphologies.^26^ In parallel, surface-tethered polymer brushes have emerged as a versatile platform for constructing hierarchical morphologies with tunable interfacial properties, enabled by the control of brush conformations and their coassembly with other polymers or particles.^27^

Anisotropic supramolecular structures have also been observed in inclusion complexes formed between α-cyclodextrin (α-CD) and poly­(ethylene oxide) (PEO)^28–30^ or poly­(ε-caprolactone) (PCL)^31^ homopolymers. Beyond PEO and PCL homopolymers, their block copolymers also form inclusion complexes with CD.^32,33^ The formation of these supramolecular structures involves specific host–guest interactions between constituent polymer blocks and CD, coupled with the self-assembly of block copolymers. Another example of supramolecular assembly involves the complexation of a host molecule, tris-*o*-phenylenedioxycycloriphosphazene (TPP), with a poly­(ethylene oxide)-*block*-poly­(octyl 4′-octyloxy-2-vinylbiphenyl-4-carboxylate) (PEO-*b*-PVBP) coil–rod block copolymer.^34^ This structure arises from host–guest interactions between PEO and TPP. This example further demonstrates the versatility of supramolecular assembly in a block copolymer system.

Herein, this study focuses on two types of 2D hybrid hairy nanostructures: irregular nanoplates and polygonal nanoplates. These 2D hybrid nanostructures were frequently observed as intermediate products during the template-mediated synthesis of methylammonium lead bromide (MAPbBr_3_) quantum nanodots using polystyrene-*block*-poly­(ethylene oxide) (PS-*b*-PEO) soft colloidal templates in TMB.^35,36^ The 2D hybrid hairy nanostructures do not exhibit photoluminescence (PL) at an emission wavelength of 365 nm and are identified as crystalline intermediates formed in hybrids of lead­(II) bromide (PbBr_2_) and PS-*b*-PEO. Furthermore, the 2D hybrid hairy nanostructures are also characterized by the presence of numerous tiny nanodots on their surfaces.^35,36^

Notably, these 2D hairy nanostructures do not form when polystyrene-*block*-poly­(2-vinylpyridine) (PS-*b*-P2VP) is used as soft templates instead of PS-*b*-PEO.^37–39^ In TMB containing PS-*b*-P2VP, PbBr_2_ complexation results only in the formation of amorphous [PbBr_3_]^−^ complexes, which are selectively encapsulated within the P2VP cores of PS-*b*-P2VP core–shell spherical micelles. The formation of these amorphous [PbBr_3_]^−^ complexes involves a hierarchical emulsion process facilitated by PS-*b*-P2VP micelles in TMB. This comparison suggests that the formation of 2D anisotropic nanostructures specifically requires the presence of PS-*b*-PEO. Furthermore, it highlights that PbBr_2_ complexation and hierarchical emulsion in TMB with PS-*b*-PEO differ fundamentally from those occurring in TMB with PS-*b*-P2VP.

To the best of our knowledge, the mechanisms underlying the formation of irregular nanosheets and polygonal nanoplates in solution and films have not yet been fully elucidated. In particular, whether the relative contents of these two nanostructures depend on the weight ratio of PS-*b*-PEO to PbBr_2_ in the hybrid system remains unclear. In this study, we demonstrate that at a high weight ratio of PS-*b*-PEO to PbBr_2_, polygonal nanoplates were predominantly observed, while irregular nanosheets were favored at lower weight ratios of PS-*b*-PEO to PbBr_2_. The 2D hairy nanostructures coexisted with worm-like nanodomains and microplates. On the contrary, the neat PS-*b*-PEO BCPs only formed worm-like nanodomains and microplates. The worm-like nanodomains originated from the simple self-assembly of complex-free PS-*b*-PEO, whereas the microplates were driven by the crystallization of the PEO block (i.e., crystallization-driven self-assembly). In contrast, irregular nanosheets and polygonal nanoplates were only obtained via BCP-assisted multiple emulsions of PbBr_2_ powder in TMB. This comparison indicates that the formation of irregular nanosheets and polygonal nanoplates requires both PS-*b*-PEO chains and inorganic complexes. The dependence of the composition on the formation of irregular nanosheets and polygonal nanoplates is detailed in the text.

## Experimental Section

### Materials

A polystyrene-*block*-poly­(ethylene oxide) (PS-*b*-PEO) block copolymer (*M*_n,PS_ = 33 kg/mol, *M*_n,PEO_ = 13.5 kg/mol, *Đ* = 1.12) was purchased from Polymer Source, Inc. TMB was obtained from Thermo Scientific, and PbBr_2_ from Sigma-Aldrich. All chemicals and the PS-*b*-PEO block copolymer were used as received without further purification.

### Preparation of Solutions and Thin Films

Twenty milligrams of PS-*b*-PEO powders was first dissolved in 1 mL of TMB and sonicated at 70 °C for 30 min to prepare BCP*_m_* solutions, with *m* denoting the quantity (i.e., 20) in units of milligrams per milliliter for the sake of brevity. After sonication, 10, 20, 50, 100, or 200 mg of PbBr_2_ powders was added to the BCP_20_ solutions to prepare precursor solutions. The precursor solutions were stirred at 800 rpm for 24 h to facilitate PbBr_2_ complexation and hierarchical emulsion formation, resulting in their turbid appearance. Finally, the turbid precursor solutions were centrifuged at 7000 rpm for 3 min to obtain supernatants and remove PbBr_2_ precipitates. For the sake of brevity, the turbid precursor solutions are denoted as t-PRE_*m*/*n*_ solutions while the centrifugated precursor supernatants are denoted as c-PRE_*m*/*n*_ solutions. The removed PbBr_2_ solids are denoted as p-PRE_*m*/*n*_ precipitates. The subscript *m*/*n* denotes weight ratio of PS-*b*-PEO to PbBr_2_. Films were prepared from the c-PRE_*m*/*n*_ solutions via drop casting on TEM grids for transmission electron microscopy (TEM) characterization or spin coating on SiO_*x*_/Si substrates for grazing-incidence small-angle scattering/wide-angle diffraction (GISAXS/GIWAXD).

### Instruments and Characterization of Materials

UV–vis absorbance spectroscopy (JASCO V-770) was performed on the precursor solutions to characterize the absorption of PbBr_2_ and its complexes. UV–vis absorbance spectra were recorded over the wavelength range of 200–600 nm at a scan speed of 400 nm/min. All of the c-PRE_*m*/*n*_ solutions were diluted at 1/10 times their original concentrations for UV–vis absorbance measurements.

Simultaneous small-angle X-ray scattering (SAXS) and wide-angle X-ray diffraction (WAXD) measurements were preformed on beamline TPS 13A of the National Synchrotron Radiation Research Center (NSRRC, Hsinchu, Taiwan). For SAXS and WAXD experiments, precursor solutions were sealed in capillary tubes (diameter of 2 mm) and characterized at 15 keV (λ = 0.827 Å). To account for background signals due to the solvent, neat TMB sealed in a capillary tube was also characterized under the same instrumentational condition. After background subtraction, sample–detector distance calibration, and absolute intensity calibration, SAXS and WAXD profiles with absolute intensities were obtained.

Films were characterized using grazing-incidence small-angle X-ray scattering (GISAXS) at beamline TLS 23A and wide-angle X-ray scattering (GIWAXD) at TPS 25A. GISAXS 2D patterns were collected at an incident angle (α_i_) of 0.13° with λ = 1.24 Å, and GIWAXD 2D patterns were collected at an α_i_ of 0.085° with λ = 0.86 Å. Thin films were prepared by spin coating at 1000 or 3000 rpm (1 min).

For morphological observations and microchemical analysis, small aliquots of precursor solutions were drop casted on TEM grids, with a filter paper placed underneath to rapidly remove the solvent. The specimens were then dried at 25 °C overnight in a vacuum oven. Low-voltage transmission electron microscopy (LV-TEM, JEOL JEM-1400) was performed at 120 kV to obtain bright-field and dark-field images for morphological observations. Selected-area electron diffraction (SAED) was conducted by using a JEOL digital system. Chemical element characterization was performed using an energy-dispersive X-ray spectroscopy (EDS). Lattice fringes were acquired at 200 kV using an ultra-high-resolution transmission electron microscope (JEOL JEM-2100F CS STEM). The removed precipitates were analyzed using in-house X-ray diffraction (Rigaku SmartLab) at 40 kV and 30 mA after drying. XRD profiles were collected in the range of 5–80° at a scan rate of 50°/min, using Cu Kα radiation (λ = 1.5406 Å). To quantitatively determine the thicknesses of the crystals, we used an atomic force microscope (AFM1000Plus, Hitachi) in tapping mode to record topographic images and height profiles. To identify PEO–Pb^2+^ interactions, Fourier transform infrared (FTIR) spectra were recorded using a Nicolet 6700 instrument (Thermo Science).

## Results and Discussion

Figure 1 shows the photos, UV–vis absorbance spectra, and SAXS profiles of the supernatants, where the initial PS-*b*-PEO/PbBr_2_ weight ratios were 20/10, 20/100, and 20/200 in 1 mL of TMB initially containing 20 mg of PS-*b*-PEO. The supernatants are turbid, indicating dispersed structures in TMB (Figure 1A). Importantly, PS-*b*-PEO is essential for dispersing PbBr_2_ in TMB. In its absence, PbBr_2_ inevitably precipitates (Figure S1A).

**Figure 1:**
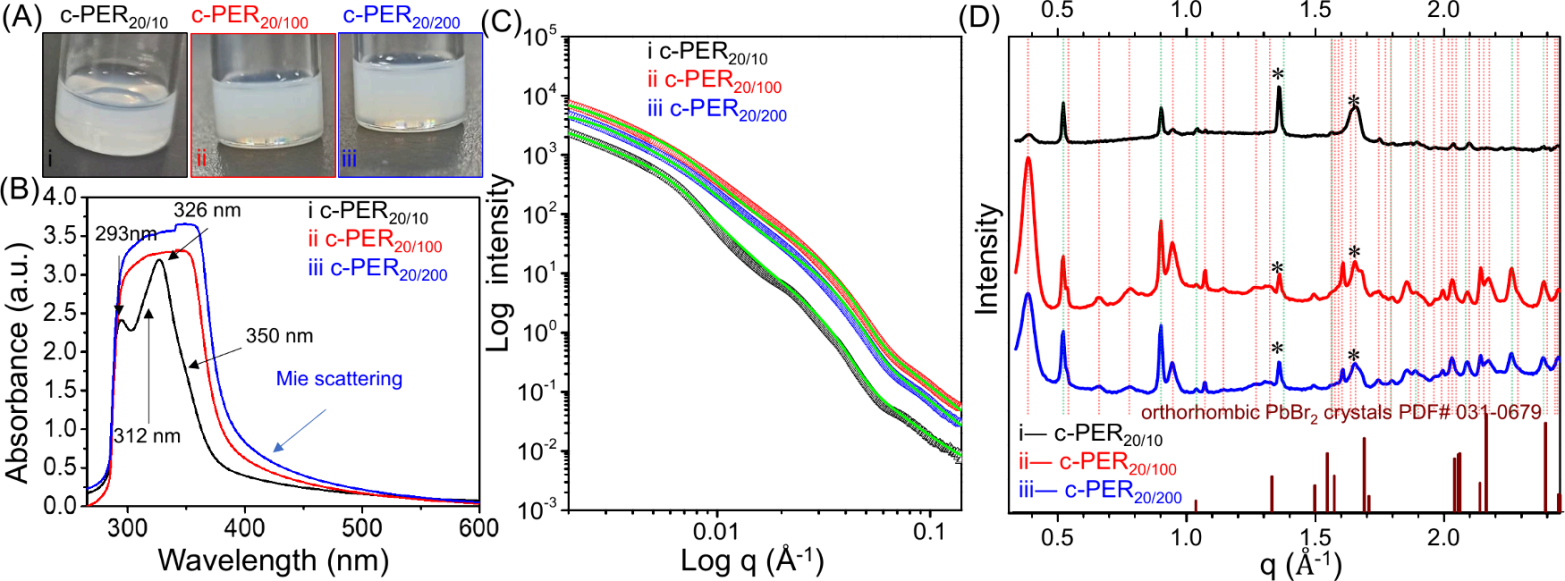
(A) Photographs, (B) UV–vis absorbance spectra, (C) SAXS profiles (symbols for experimental data and green lines for fitted curves), and (D) WAXD profiles collected for (i) c-PRE_20/10_, (ii) c-PRE_20/100_, and (iii) c-PRE_20/200_ solutions in the dried state. In panel D, the stick pattern represents the orthorhombic PbBr_2_ crystal (PDF #031-0679). The diffraction peaks corresponding to a hexagonal complex crystal with *P*6*mm* symmetry are highlighted by green lines, those corresponding to an orthorhombic complex crystal with *Cmca* symmetry by red lines, and the diffraction peaks associated with the PEO crystal by asterisks.

The turbid supernatants were further analyzed by UV–vis absorbance spectroscopy to identify complex species. When *m*/*n* = 20/10, the UV–vis absorbance spectrum exhibits four absorption bands at approximately 293, 312, 326, and 350 nm (Figure 1B_i_). These bands are attributed to the combined absorbance contributions from the PS-*b*-PEO/PbBr_2_, [PbBr_6_]^4–^, [PbBr_3_]^−^, and [PbBr_4_]^2–^ complexes. The assignment of the absorbance bands associated with PbBr_2_ and its complexes is based on previous studies.^36,40,41^ When *m*/*n* = 20/100 and 20/200, the absorbance bands intensify and reach the detector’s limit, resulting in a broad truncation effect (Figure 1B_ii_,B_iii_). This truncation arises from the presence of highly concentrated solutes that strongly absorb light, causing excessive attenuation of the transmitted signal, which has been discussed by Junisu et al.^42^ By contrast, these spectral features were absent in control solutions containing only PbBr_2_ without PS-*b*-PEO (Figure S1B), confirming that PbBr_2_ complexation in TMB occurs only in the presence of PS-*b*-PEO.

Figure 1C displays the corresponding SAXS profiles of the c-PRE_*m*/*n*_ solutions. Different from the SAXS profiles of a BCP_20_ solution measured at 25 °C (Figure S2A) and 80 °C (Figure S2C), all SAXS curves shown in Figure 1C exhibit multiple Guinier and Porod-like scattering features, indicative of hierarchical or multiple structures. It has been well demonstrated that the multiple Guinier and Porod-like scattering features of hierarchical structures can be well fitted by the Beaucage model.^43,44^ To quantitatively analyze these structural details, we performed two-level Beaucage model fitting, with the fitted lines overlaid on the experimental SAXS data in Figure 1C for comparison. The fitted lines were based on the structural parameters listed in Table S1. The perfect agreement between the fitted and experimental curves validates the hierarchical nature of the hybrid nanostructures formed by PS-*b*-PEO and PbBr_2_.

Figure 1D presents the WAXD profiles of the supernatants at different PbBr_2_/PS-*b*-PEO weight ratios. The diffraction peaks at *q* = 1.36 and 1.643/1.647 Å^–1^ correspond to the (120) and (112)/(032) planes of PEO monoclinic crystals, respectively, which is consistent with the literature.^45,46^ These peaks also appear in the WAXD profile of a BCP_20_ solution at 25 °C (Figure S2B) but disappear completely at 80 °C (Figure S2D), confirming their origin from PEO crystallites. In the PbBr_2_/PS-*b*-PEO hybrid systems, the intensities of these PEO diffraction peaks decrease as the PbBr_2_ concentration increases, suggesting that a higher PbBr_2_ content may suppress the crystallization of PEO.

In addition to the characteristic PEO diffractions, several additional diffraction peaks emerge in the WAXD profiles. Notably, the low-*q* region (*q* < 1.1 Å^–1^) exhibits several intense diffractions that cannot be assigned to either PEO monoclinic crystals or PbBr_2_ orthorhombic crystals (stick pattern in Figure 1D).

Figure S3 demonstrates the in-house WAXD profiles of precipitates separated from the supernatants. Comparing Figure 1D and Figure S3 demonstrates that the appearance of these additional diffraction peaks suggests the formation of alternative crystalline complex structures, distinct from both PEO monoclinic crystals and PbBr_2_ orthorhombic crystals. The alternative complex structures exhibit two series of diffractions in Figure 1D. First, some diffraction peaks (marked by green lines in Figure 1D) located at positions with a *q* ratio of 1/3^1/2^/4^1/2^/7^1/2^/9^1/2^/12^1/2^/13^1/2^/16^1/2^/19^1/2^/21^1/2^ in series with the peak at 0.522 Å^–1^ agree with the results of Chung et al.^36^ This *q* ratio indicates that one of the alternative crystalline structures has hexagonal symmetry (Table S2).

In contrast, the second series of diffraction peaks (red lines in Figure 1D) shows a broad shape. We found that the diffraction peaks of the second series can be assigned to an orthorhombic complex crystal with *Cmca* symmetry (see Table S2).^36^ Figure 1D demonstrates that the c-PRE_20/10_ solution primarily contained nanostructures having hexagonal complex crystals with *P*6*mm* symmetry while the c-PRE_20/100_ and c-PRE_20/200_ solutions contained nanostructures having orthorhombic complex crystals with *Cmca* symmetry.

To clarify structural details further, we performed low-voltage TEM and ED characterization for morphological observations. To this end, 10 μL portions of the supernatants were dropped on TEM grids, which were placed on top of filter paper. With filter paper, most solvent could be quickly removed, and only structures were left on TEM grids. The drop-casted samples were dried in a vacuum at 25 °C (overnight) to remove residual solvent.

Figure 2 shows the TEM and ED results of the hybrids. Scrutiny of the TEM images reveals that the hairy 2D nanostructures exhibit two distinct morphologies: irregular nanosheets and polygonal nanoplates (Figure 2). These 2D nanostructures inevitably coexist with worm-like nanodomains and PS-*b*-PEO crystallites in the hybrids (Figures S4–S6) but are completely absent from neat PS-*b*-PEO (Figure S7). Furthermore, the contents of irregular nanosheets and polygonal nanoplates depend on the PS-*b*-PEO/PbBr_2_ weight ratios, for which a weight ratio of 20/10 favors the major formation of polygonal nanoplates but the minor coexistence of irregular nanoplates (Figure 2A,B). In comparison, weight ratios of 20/100 and 20/200 preferentially grow irregular nanosheets (Figure 2D,E). As a result, the content of irregular nanosheets is more dominant than that of polygonal nanoplates. Nevertheless, the contents of irregular nanosheets and polygonal nanoplates are not obviously influenced by prolonged stirring (Figure S8). This comparison indicates that these structures are thermodynamically stable rather than kinetically trapped. To determine a critical concentration of PbBr for dominantly forming irregular nanosheets, we additionally prepared two more samples with ratios of 20/20 and 20/50 for TEM characterization in addition to the three original samples (20/10, 20/100, and 20/200). Figure S9 indicates that beyond a certain PbBr_2_ concentration irregular nanosheets become the dominant 2D morphology.

**Figure 2:**
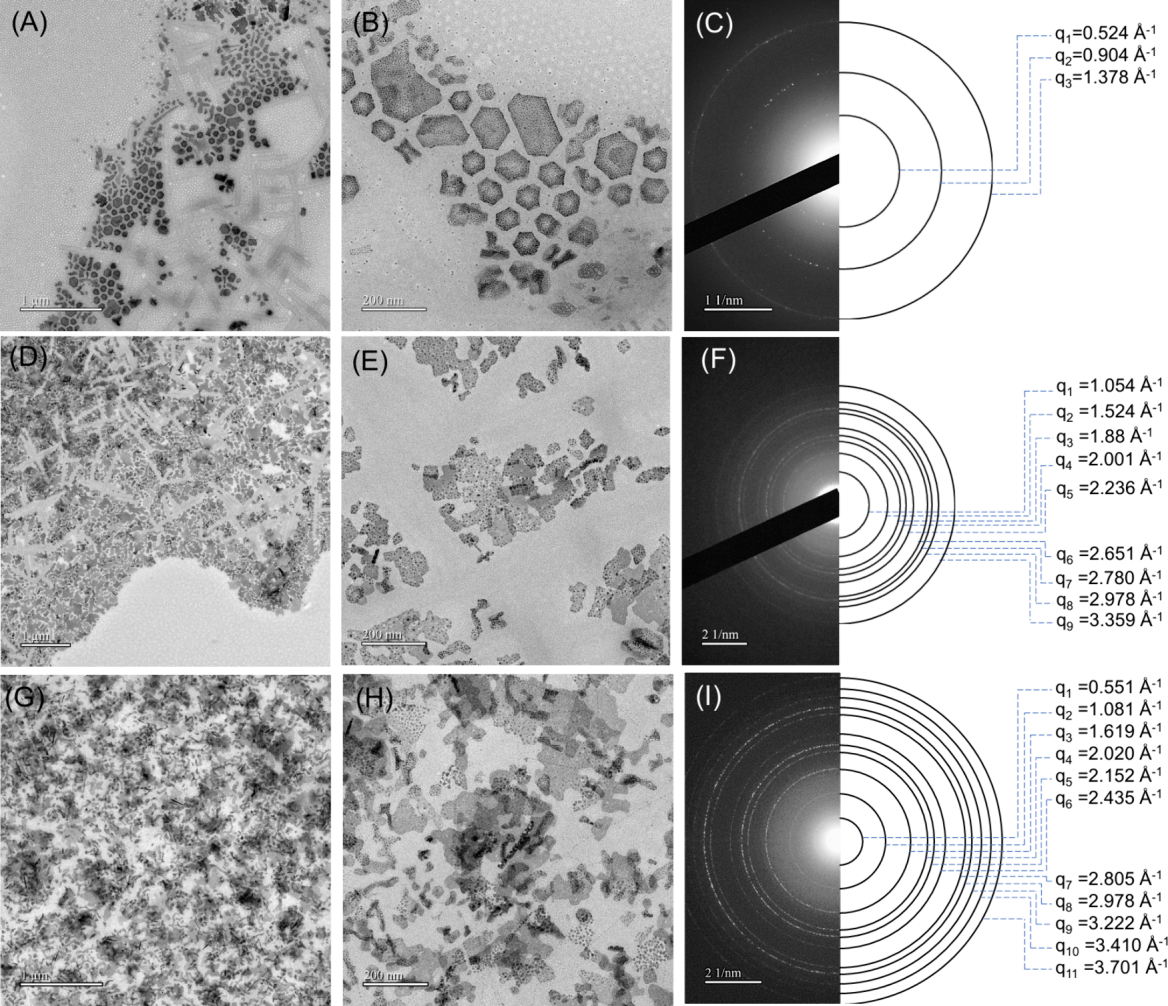
(A, D, and G) Low-magnification and (B, E, and H) high-magnification bright-field TEM images and (C, F, and I) ED patterns collected for c-PRE_20/10_ (top row), c-PRE_20/100_ (middle row), and c-PRE_20/200_ (bottom row) solutions in a dried state.

The ED pattern of a selected aggregate of polygonal nanoplates shows three powder rings located at 0.524, 0.904, and 1.378 Å^–1^ (Figure 2C). The positional ratio of the powder rings is 1/3^1/2^/7^1/2^ with respect to the first ring at 0.524 Å^–1^. The ratio indicates that the polygonal nanosheets have hexagonal arrays with *P*6*mm* symmetry.

In contrast, the ED pattern of irregular nanosheets exhibits numerous powder rings, located at 0.551, 1.081, 1.619, 2.020, 2.152, 2.435, 2.805, 2.978, 3.222, 3.410, and 3.701 Å^–1^, which cannot be assigned to the *P*6*mm* symmetry (Figure 2F,I). This discrepancy indicates that the irregular nanosheets are comprised of another type of crystals. Notably, the surfaces of the irregular nanosheets and polygonal nanoplates are covered with numerous tiny PbBr_2_ nanodots (Figure S10). Quantitative AFM analysis shows that irregular nanosheets have an average thickness of ∼25 nm, while polygonal nanoplates have an average thickness of ∼31 nm (Figure S11). These values should be regarded as approximate as the decoration of tiny PbBr_2_ nanoparticles on their surfaces can locally increase the measured thickness.

EDS analysis was selectively performed on a single irregular nanosheet and a single polygonal nanoplate chosen from the TEM images (Figure 3A,C). Panels B and D of Figure 3 illustrate the spatial distribution of elements, showing that the irregular nanosheet contains more Pb and Br than does the polygonal nanoplate. Consequently, the polygonal nanoplate has more O than does the irregular nanosheet. These differences in Pb and Br concentrations likely influence the assembly pathways, with higher PbBr_2_ concentrations likely favoring the formation of irregular nanosheets and lower PbBr_2_ concentrations likely favoring polygonal nanoplates.

**Figure 3:**
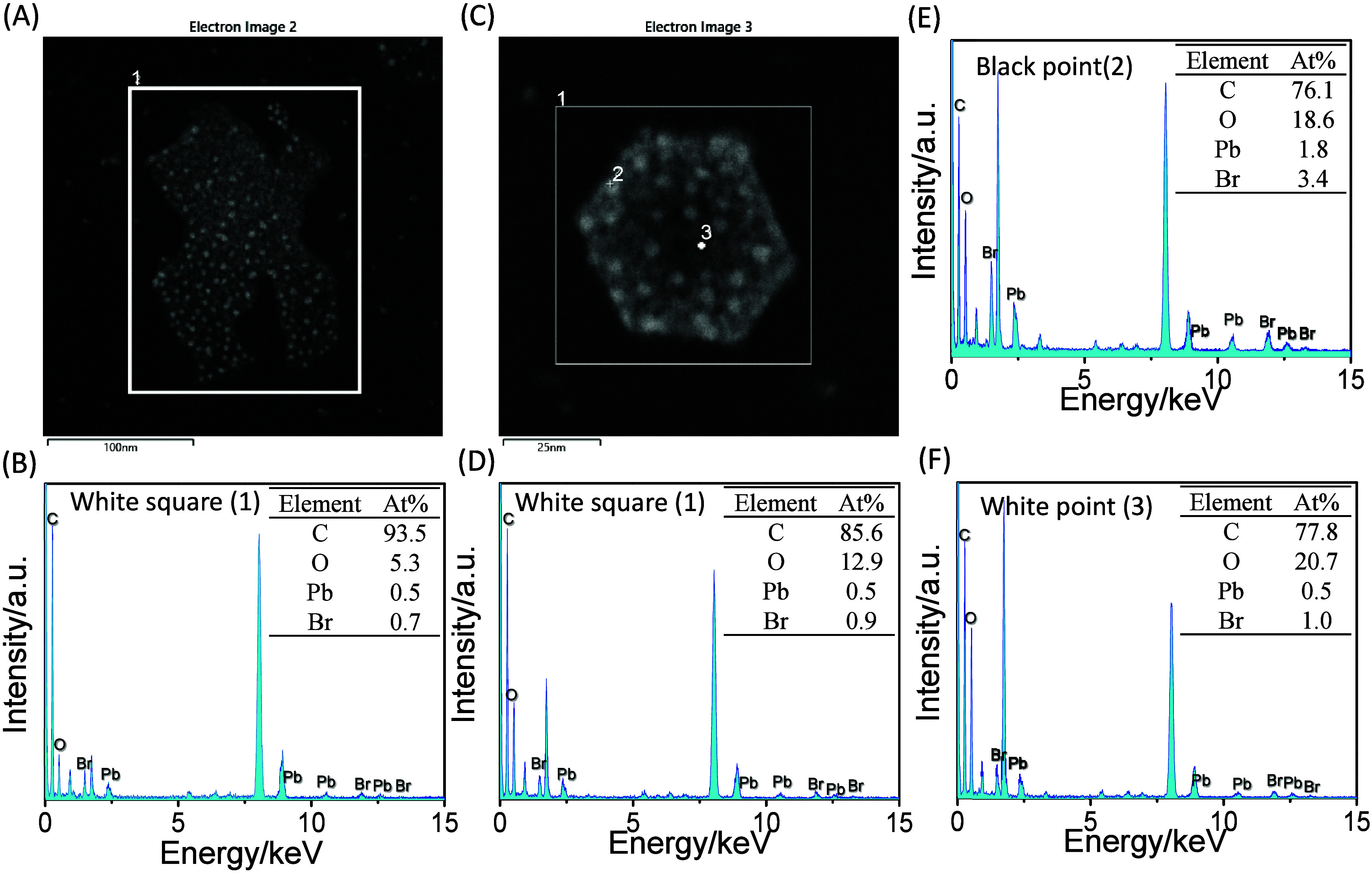
(A and C) High-angle annular dark-field TEM images and (B and D–F) EDS images collected on a selected (A and B) irregular nanosheet and (C–F) polygonal nanoplate. The TEM images and EDS profiles were collected on the c-PRE_20/10_ solution in a dried state.

Furthermore, we characterized the chemical compositions of local areas on the polygonal nanoplate (Figure 3E,F). Interestingly, variations in Pb and Br contents were observed, with tiny nanodots (Figure 3E) showing higher Pb and Br levels but lower O contents compared to nanodot-free areas (Figure 3F). Quantitative analysis of panels B and D of Figure 3 suggests that the [EO]/[Pb^2+^] molar ratio is likely higher for polygonal nanoplates (∼25/1) than for irregular nanosheets (∼10/1). These values are comparable to those of Huq et al.^47^ and Wendsjö et al.^48^ for PEO complexes with heavy metal halides. While this EDS-based approach is only semiquantitative and may be influenced by residual oxygen species, unlike NMR-based methods,^49^ it still provides an approximate estimate of coordination density. Notably, the [Br^–^]/[Pb^2+^] ratio in polygonal nanoplates is approximately 2/1, which matches the stoichiometry of PbBr_2_ and likely supports the reliability of the EDS-derived composition.

Figure 4 schematically illustrates the formation of polygonal nanoplates and irregular nanosheets under stirring in TMB with added PS-*b*-PEO, along with their surface decoration by capped PbBr_2_ nanodots. The development of these anisotropic hairy 2D nanostructures involves a sequence of processes, including multiple emulsions, PbBr_2_ fragmentation, capping of PbBr_2_ fragments through surface adsorption of PS-*b*-PEO chains, PbBr_2_ dissociation and complexation, host–guest interactions between PEO chains and PbBr_2_-based complexes, inclusion crystallization, and the final adsorption of capped PbBr_2_ nanodots to the surface.

**Figure 4:**
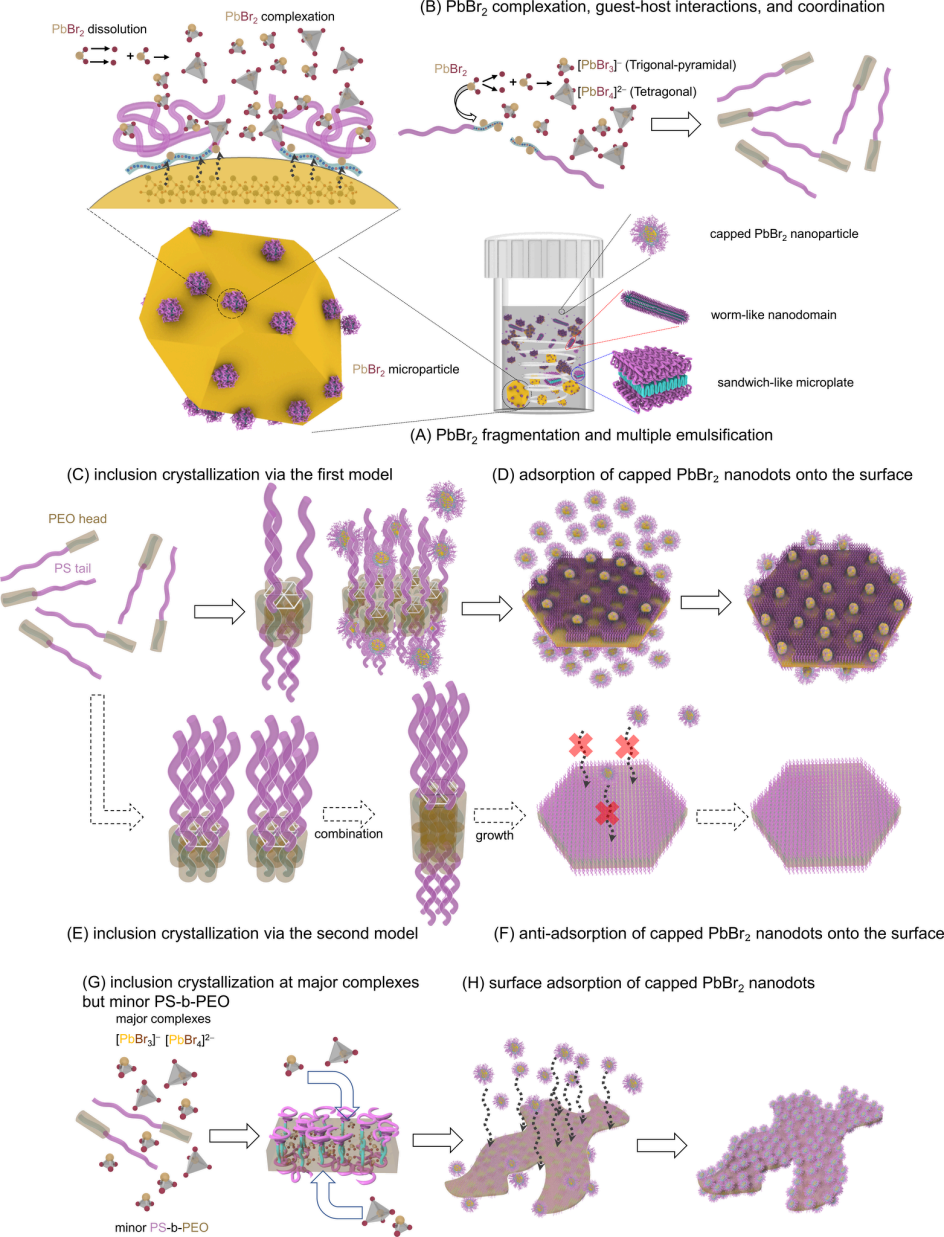
Schematic illustration of the formation pathways for polygonal nanoplates and irregular nanosheets in TMB containing PS-*b*-PEO under stirring. (A) PbBr_2_ fragmentation and multiple emulsification. (B) PbBr_2_ complexation, guest–host interactions, and coordination. (C) Inclusion crystallization via the first model. (D) Adsorption of capped PbBr_2_ nanodots to the surface. (E) Inclusion crystallization via the second model. (F) Antiadsorption of the capped PbBr_2_ nanodots to the surface. (G) Inclusion crystallization at major complexes but minor PS-*b*-PEO. (H) Surface adsorption of capped PbBr_2_ nanodots. For the sake of clarity, worm-like nanodomains and sandwich-like microplates are not depicted in panels B–H.

In TMB with added PS-*b*-PEO, PbBr_2_ microparticles are initially adsorbed by PS-*b*-PEO chains. These PS-*b*-PEO-adsorbed PbBr_2_ microparticles become dispersed through microscale emulsions in the TMB (Figure 4A). Furthermore, stirring promotes fragmentation of some PbBr_2_ microparticles into smaller PbBr_2_ fragments. These smaller fragments are more readily adsorbed by PS-*b*-PEO chains, leading to the formation of nanoscale emulsions. At the interfaces between PEO and PbBr_2_, dissociation and complexation of PbBr_2_ occur, resulting in the formation of [PbBr_3_]^−^ and [PbBr_4_]^2–^ complexes. This process is driven by the strong interactions between Pb^2+^ cations and two lone pairs of electrons present on each ether group in PEO (Figure 4A). Such PEO–Pb^2+^ coordination is supported by our FTIR spectra (Figure S12). This observation is consistent with previous studies showing that electron-rich ether oxygens can act as oxygen-donor ligands and coordinate with Pb­(II) centers.^50,51^

Furthermore, the formation of complex crystals necessarily alters the equilibrium between unimers and micelles in TMB. Prior to the addition of PbBr_2_, PS-*b*-PEO unimers and micelles coexist in equilibrium, consistent with earlier reports.^52,53^ Complex crystal formation primarily involves the participation of PS-*b*-PEO unimers rather than assembled mesostructures in coordinating with [Pb*_x_* Br_*y*_]^2^ *^x^* ^–^ *^y^* complexes. Because PS-*b*-PEO assemblies are typically tens of nanometers in size, they are too large to be directly incorporated into the crystal lattice. In contrast, unimers with accessible PEO chains can effectively coordinate with Pb^2+^ species, thereby contributing to nucleation and growth. We therefore propose that complex crystal formation preferentially consumes free PS-*b*-PEO unimers, which coexist with micelles before PbBr_2_ addition. At the same time, tiny PbBr_2_ nanoparticles may be encapsulated within PS-*b*-PEO nanodomains, providing another route of stabilization. Both mechanisms perturb the thermodynamic balance. As unimers are consumed through complexation, additional unimers are released from micellar assemblies to restore equilibrium.

When *m*/*n* = 20/10, where PS-*b*-PEO is the major component and [PbBr_3_]^−^ and [PbBr_4_]^2–^ complexes are less abundant, the selective binding of Pb^2+^ cations to PEO chains increases the rigidity of the cation-bound PEO chains compared to the PS chains and enhances the strength of segregation between PS and PEO. Such salt-induced enhancement of segregation strength has also been reported in PS-*b*-PEO/lithium salt hybrids.^54–57^ Consequently, each PS-*b*-PEO chain behaves like a rod–coil chain or a head–tail molecular surfactant (Figure 4B). If the PEO block bound to the complexes is considered a rigid rod (or head) and the complex-free PS block is treated as a flexible coil (or tail), then the packing involves side-by-side interactions between the PEO rods, forming a hydrophilic core.

These interactions can adopt two possible orientations. In the first, PS coils extend in one direction away from the core due to solvent swelling. In the second, PS coils extend in the opposite direction. While the PEO heads remain within the core, this dual configuration allows for efficient molecular packing, which may promote the formation of 2D hexagonal crystals by accommodating two distinct arrangements of the building blocks (Figure 4C).

For this side-by-side packing, both surfaces of each polygonal nanoplate are covered by a single layer of loosely packed PS chains. As a result, the weak steric repulsion from these loose PS chains allows for the surface decoration of the PbBr_2_ nanodots. Furthermore, each PEO core experiences a minimal entropy penalty since both sides are capped by a single layer of PS chains. The surface deposition of tiny capped PbBr_2_ nanodots can further reduce the enthalpic penalty (Figure 4D).

Unlike the dual orientations proposed in the first model, the second model suggests that PS chains extend in a single direction from the core, either entirely upward or downward, covering only one side of the polygonal core, while the other side remains exposed to TMB without a PS capping layer (Figure 4E). This packing has three key implications.

First, the exposed side, composed of PEO bound with complexes, experiences a high enthalpic penalty due to direct exposure to TMB. To minimize this unfavorable energy state, the formation of a thicker structure through the combination of two hexagonal arrays would be required. In this case, the core should be thicker to maintain the structural stability (Figure 4E). Second, the swollen PS chains generate strong steric repulsion, preventing the surface decoration of the PbBr_2_ nanodots (Figure 4F). Third, this packing promotes the formation of 3D hexagonal crystals with long-range order and layer-by-layer stacking, which would likely result in diffraction signals in the SAXS region.

Based on morphological observations and structural characterizations, we believe that the first model of hexagonal packing is more likely to account for the formation of 2D hairy polygonal nanoplates decorated with abundant PbBr_2_ nanoparticles compared to the second model. The absence of SAXS diffraction signals further supports this conclusion. The second model of hexagonal packing might also account for the formation of polygonal nanoplates. However, we believe that this probability of the second model should be much lower than that of the first model.

Hexagonal crystals with *P*6*mm* symmetry have been frequently observed in a broad range of hybrid systems, underscoring the generality of this mesophase motif and its characteristic packing in materials formed via supramolecular, coordination-driven, or inclusion crystallization self-assembly processes.^29–32,58–60^ Our observation of *P*6*mm*-symmetric hexagonal crystals formed from majority PS-*b*-PEO chains and minority PbBr_2_-based complexes is thus consistent with these findings and highlights the versatile role of soft templating and ligand coordination in directing hexagonal symmetry across diverse hybrid nanostructures.

When *m*/*n* = 20/100 and 20/200, where [PbBr_3_]^−^ and [PbBr_4_]^2–^ complexes are dominant and PS-*b*-PEO is the minor component, inclusion crystallization instead favors the formation of irregular nanosheets (Figure 4G). These nanosheets adopt an orthorhombic complex crystal with *Cmca* symmetry. The shift from polygonal nanoplates to irregular nanosheets suggests that higher PbBr_2_ concentrations promote the formation of larger anisotropic structures. Salt-induced phase transitions and the composition-dependent tunability of domain size have been frequently found in hybrids of PEO-based block copolymers with lithium salts.^61–63^

In our system, Figure S9 shows that polygonal nanoplates dominate at lower PbBr_2_ contents (10–20 mg mL^–1^), whereas irregular nanosheets become predominant at higher PbBr_2_ loadings (≥50 mg mL^–1^). Importantly, both morphologies coexist even at high PbBr_2_ concentrations, though the fraction of polygonal nanoplates decreases markedly and may not be easily identified by TEM (Figure 2D,E,G,H and Figures S8C,D and S9E–J). This coexistence is corroborated by WAXD results (Figure 1D), which display diffraction peaks characteristic of both complex crystals.

These findings suggest that the formation of polygonal nanoplates and irregular nanosheets is governed by the relative availability of Pb^2+^ cations and [Pb_*x*_Br_*y*_]^2*x*−*y*^ complexes that coordinate with the PEO chains. At low PbBr_2_ concentrations, coordination between Pb^2+^ and the ether groups of PEO is favored due to the lower abundance of [Pb_*x*_Br_*y*_]^2*x*−*y*^ complexes, producing discrete polygonal nanoplates through inclusion crystallization self-assembly (Figure 4B,C). As the PbBr_2_ concentration increases, the higher concentration of [Pb_*x*_Br_*y*_]^2*x*−*y*^ complexes promotes their cocoordination with PEO, leading to the formation of irregular nanosheets via inclusion crystallization self-assembly (Figure 4G).

Furthermore, the irregular nanosheets also have surfaces enriched with abundant BCP-capped PbBr_2_ nanodots. The decoration of tiny nanodots on the surfaces of hairy nanosheets and nanoplates is driven by the self-assembly of capped PbBr_2_ nanoparticles (Figure 4D, H). Such nanoparticle decoration is known to depend on noncovalent interactions^64^ or the grafting density of surface ligands.^65^ In our case, however, the coated PbBr_2_ nanoparticles (NPs) do not adsorb to the surfaces of PS-*b*-PEO microsquares with a sandwich-like structure. The absence of BCP-capped PbBr_2_ NPs on the microsquare surfaces suggests repulsive interactions between the PS shells of the microsquare single crystals and those of the capped PbBr_2_ NPs. This observation raises the important question of why, in contrast, the surfaces of irregular nanosheets and polygonal nanoplates adsorb BCP-capped PbBr_2_ NPs (manifested as PbBr_2_ nanodots). We propose that this discrepancy arises from differences in tethering density between the microsquare single crystals and the irregular nanosheets/polygonal nanoplates. Our working hypothesis is as follows.

According to previous studies,^64–67^ we believe that microsquare single crystals have compact PEO cores, each sandwiched by PS shells with a high tethering density. The tightly packed PS shells adopt a brush conformation, imparting significant steric repulsion that prevents the adsorption of BCP-capped PbBr_2_ NPs. In contrast, the formation of irregular nanosheets and polygonal nanoplates involves the inclusion of [PbBr_3_]^−^ and [PbBr_4_]^2–^ complexes within the PEO chains. This inclusion disrupts the packing of the PS shell-forming chains, resulting in a mushroom-like conformation of the PS shells. The loosened packing of the PS chains reduces steric repulsion, allowing the BCP-capped PbBr_2_ NPs to easily adsorb to the surfaces of irregular nanosheets and polygonal nanoplates (Figure 4D,H).

The precursor solutions were spin-coated (1000 rpm for 1 min) onto silicon wafers for GISAXS and GIWAXD characterization. The 2D GISAXS patterns exhibit intense diffuse scattering and two parallel Yoneda streaks (Figure 5A). The diffuse scattering is anisotropically elongated in the *q*_⊥_ direction, which is typically characteristic of anisotropic textures. Only form-factor scattering is observed, with no evident structure-factor diffraction. In other words, truncation rods and diffraction spots, which are typically associated with the long-range spatial ordering of nanodomains and nanostructures, are absent. Their absence indicates that the 2D hairy nanostructures, nanocylinders, and perforated layers lack long-range spatial order and periodicity in the dried state.

**Figure 5:**
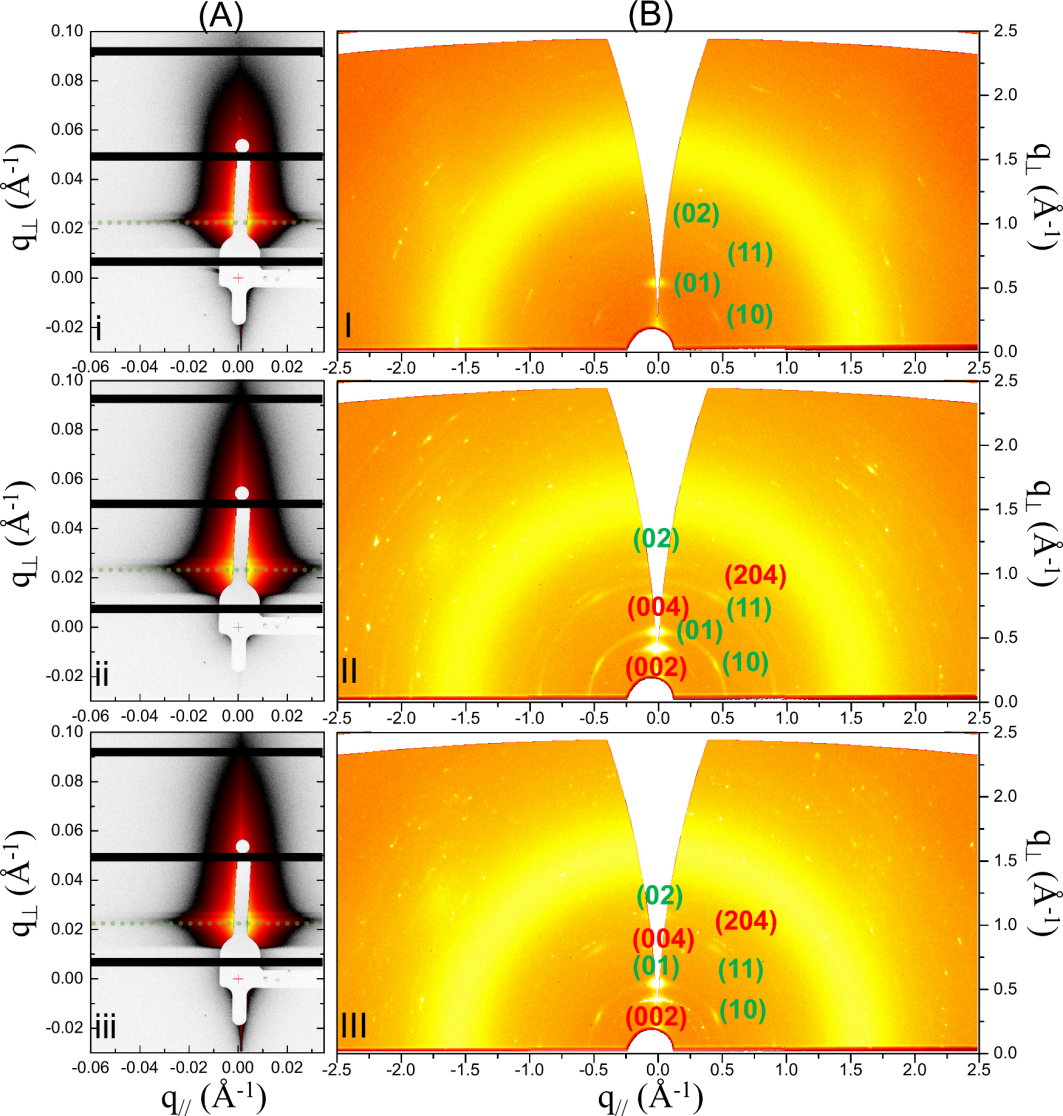
(A) 2D GISAXS patterns and (B) 2D GIWAXD patterns of films spin-coated from the c-PRE_*m*/*n*_ solutions with *m*/*n* ratios of (i and I) 20/10, (ii and II) 20/100, and (iii and III) 20/200. Reflections are indexed for hexagonal complex crystals and orthorhombic complex crystals, respectively.

In comparison, the GIWAXD 2D patterns show three features (Figure 5B). The 2D GIWAXD patterns exhibit three key features. First, an intense halo is observed, which is attributed to the amorphous PS-*b*-PEO matrix. Second, symmetric diffraction spots appear on both sides of *q*_∥_ = 0 particularly in a low-*q* region defined by *q*_⊥_ < 1.1 Å^–1^ and *q*_∥_ < 1.1 Å^–1^, indicating the presence of well-aligned 2D nanostructures. Third, small speckles are randomly scattered throughout the GIWAXD patterns without a symmetric placement. Moreover, the speckles cannot be well assigned to *P*6*mm* and *Cmca* symmetries (Figure S13).

The spin-coated films exhibit GIWAXD fiber patterns, indicating that the nanostructures possess an in-plane random orientation but maintain a preferential alignment along the substrate’s normal direction (Figure 5B). The spin-coated film prepared with an initial *m*/*n* ratio of 20/10 shows a GIWAXD pattern (Figure 5B_I_) with diffraction spots (marked by green circles) located at *q* values of 0.52, 0.90, and 1.08 Å^–1^. The positions correspond to a series of *q*/q* ratios of 1/3^1/2^/4^1/2^ with respect to q* = 0.52 Å^–1^. The diffraction spots correspond to (01), (11), and (02) reflections, respectively, of hexagonal complex arrays with *P*6*mm* symmetry (Figure S13A). The GIWAXD pattern also shows that hexagonal complex crystals likely adopt a parallel orientation on SiO_*x*_/Si.

In contrast, the spin-coated films prepared with higher *m*/*n* ratios of 20/100 and 20/200 exhibit GIWAXD fiber patterns featuring additional diffractions (Figure 5B_II_,B_III_) in addition to the diffractions of hexagonal complex crystals. These additional diffractions, located at *q* values of 0.39, 0.79, and 0.95 Å^–1^, respectively, cannot be attributed to hexagonal arrays with *P*6*mm* symmetry. Instead, the additional diffractions can be assigned to (002), (004), and (204) reflections, respectively, of orthorhombic complex crystals with *Cmca* symmetry (Figure S13B,C).

Note that the GIWAXD patterns primarily show the intense symmetric diffractions at the low-*q* region (*q*_∥_ and *q*_⊥_ both less than 1.1 Å^–1^) as compared to the WAXD patterns of the precursor solutions. Furthermore, the GIWAXD patterns show no diffractions associated with PEO crystallites, suggesting that the spin coating can remove crystalline PS-*b*-PEO microplates. This observation suggests that interfacial effects, rather than density, govern the removal of microplates. Their higher interfacial activity likely renders them more susceptible to elimination during spin coating. In addition, several diffractions in the high-*q* region (>1.1 Å^–1^) of WAXD are weak and appear as random speckles if they exist in the same region of GIWAXD.

Figure 6 schematically illustrates the spatial orientations for irregular nanosheets and polygonal nanoplates in films deposited on SiO_*x*_/Si by spin coating. Figure 6 is based on the GISAXS and GIWAXD characterization shown in Figure 5. When deposited onto silicon substrates by spin coating, polygonal nanoplates predominantly adopt an edge-on (i.e., standing) orientation (Figure 6A,B) while irregular nanosheets adopt a face-on (i.e., lie down) orientation (Figure 6D). These orientations are primarily influenced by the spin-coating process. In contrast, drop-casting results in random orientations for both irregular nanosheets and polygonal nanoplates.^68^

**Figure 6:**
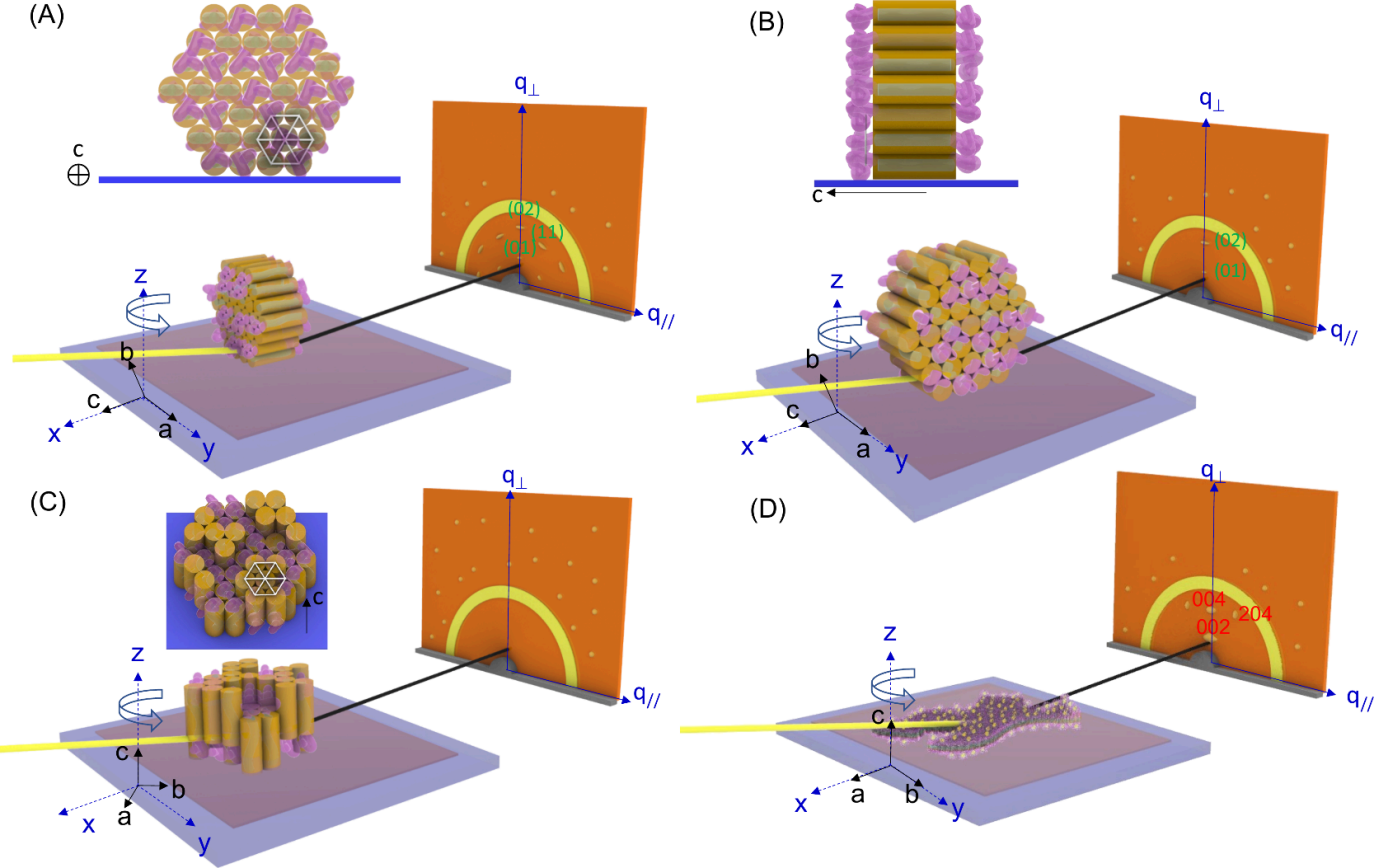
Schematic illustrations of real and reciprocal space (i.e., GIWAXD) of (A and B) edge-on and (C) face-on polygonal nanoplates composed of hexagonal complex crystals and (D) face-on irregular nanosheets composed of orthorhombic complex crystals. In GIWAXD, three types of signals are observed: diffuse rings, symmetric diffractions, and random speckles. For the sake of simplicity, worm-like nanodomains and microplates are ignored in the schema because those structures are driven by incompatibility- or crystallization-driven self-assembly of neat PS-*b*-PEO.

Polygonal nanoplates in the edge-on orientation are kinetically trapped in a metastable state, possessing a slightly higher potential energy than their face-on counterparts. However, due to their small sizes, the edge-on orientation remains more favorable for polygonal nanoplates. The presence of faceted edges further facilitates their proper deposition. With the edge-on orientation, the hexagonal crystals within the edge-on polygonal nanoplates favor a parallel orientation, thus resulting in the observed GIWAXD pattern (Figures 5B_I_ and 6A,B). Nevertheless, the edge-on polygonal nanoplates have a random in-plane orientation. The random in-plane orientation of polygonal nanoplates results in intense (01) and (02) diffractions but weak (11) diffraction (Figure 5B_I_).

There should be polygonal nanoplates with a face-on orientation in spin-coated films, although the GIWAXD characterization does not capture such diffractions associated with face-on polygonal nanoplates (Figure 6C). If polygonal nanoplates were to adopt a face-on orientation, the PS chains in direct contact with the hydrophilic silicon substrate would experience slight compression. This compression may induce both enthalpic and entropic penalties, enthalpic penalties arising from unfavorable PS–silicon interactions and entropic penalties resulting from constrained PS chains at the SiO*_x_*/Si interface. Additionally, the patchy surface coverage of compressed PS chains could destroy crystal packing, whereas standing polygonal nanoplates are less likely to experience such effects. This reason explains why the GIWAXD 2D patterns show intense diffractions for edge-on polygonal nanoplates but no diffractions for face-on polygonal nanoplates.

In contrast, irregular nanosheets, characterized by irregular edges, are unlikely to adopt an edge-on orientation on SiO*_x_*/Si (Figure S14A). Although the face-on orientation may also introduce enthalpic and entropic penalties, these effects are likely compensated by a reduction in the potential energy due to gravity (Figure 6D and Figure S14B). Given that irregular nanosheets incorporate a higher fraction of [PbBr_3_]^−^ and [PbBr_4_]^2–^ complexes, their PEO cores are expected to be relatively rigid and resistant to deformation. This rigidity further mitigates structural distortions caused by the patchy surface coverage of the compressed PS chains. With the flat-on orientation, the orthorhombic complex crystals orient with their *c* axis being parallel to the normal direction of the film (Figures 6D).

## Conclusions

We have demonstrated the formation of irregular nanosheets and polygonal nanoplates through the interaction of PS-*b*-PEO and PbBr_2_ in TMB. These 2D nanostructures feature surfaces embedded with numerous PbBr_2_ nanodots, each capped with a thin layer of PS-*b*-PEO via surface adsorption. Unlike conventional PS-*b*-PEO self-assembly, their formation is driven by a combination of PbBr_2_ complexation, host–guest interactions, and inclusion crystallization.

PEO, with its ether groups capable of strongly binding Pb^2+^, promotes PbBr_2_ dissolution and complexation, leading to the formation of [PbBr_3_]^−^ and [PbBr_4_]^2–^ complexes in TMB. These complexes undergo inclusion crystallization with PEO chains, forming irregular nanosheets and polygonal nanoplates. EDS analysis confirms that [PbBr_3_]^−^ and [PbBr_4_]^2–^ complexes dominate the irregular nanosheets, while PS-*b*-PEO is present at low levels. WAXD and ED analyses reveal that the irregular nanosheets adopt an orthorhombic structure (*Cmca* symmetry) with the following unit cell parameters: *a* = 2.353 nm, *b* = 0.420 nm, and *c* = 3.322 nm.

Similarly, polygonal nanoplates are primarily composed of PS-*b*-PEO, with minor amounts of [PbBr_3_]^−^ and [PbBr_4_]^2–^ complexes, as confirmed by EDS. WAXD and ED indicate a hexagonal structure (*P*6*mm* symmetry) with *a*_hex_ = 1.395 nm and γ = 120°. The inclusion of PbBr_2_-based complexes within PEO disrupts the packing of the PS shell-forming chains, leading to a mushroom-like conformation of the PS shells. This weakens steric repulsion, facilitating the adsorption of BCP-capped PbBr_2_ nanoparticles to the surfaces of irregular nanosheets and polygonal nanoplates.

In spin-coated films, irregular nanosheets and polygonal nanoplates adopt different orientations, where an edge-on orientation is kinetically favored for polygonal nanoplates, whereas a face-on orientation is thermodynamically favored for irregular nanosheets.

Our findings provide several insights into the formation of 2D hybrid nanostructures via a unique combination of surface adsorption, PbBr_2_ dissolution and complexation, host–guest interactions, and inclusion crystallization. This work paves the way for the further exploration of polymer-assisted perovskite synthesis and the development of tailored hybrid materials.

## Supplementary Material



## Notes

Photographs and UV–vis absorbance spectra of control solutions (Figure S1); structural parameters used for SAXS fitting (Table S1); SAXS and WAXD profiles of a BCP_20_ solution (Figure S2); in-house WAXD profiles of p-PRE_*m*/*n*_ precipitates (Figure S3); Miller indices, *q* values, *d* spacings, and *q* ratios (Table S2); bright-field TEM images and ED patterns of worm-like nanodomains formed in c-PRE_*m*/*n*_ solutions (Figure S4); bright-field TEM images and EDS profiles of worm-like nanodomains (Figure S5); bright-field TEM image collected for a t-PRE_20/10_ solution (Figure S6); bright-field TEM images of a BCP_20_ solution (Figure S7); low- and high-magnification bright-field TEM images of dried samples prepared by prolonged stirring (Figure S8); bright-field TEM images of c-PRE_*m*/*n*_ solutions (Figure S9); bright-field and high-angle annular dark-field images of irregular nanosheets and polygonal nanoplates (Figure S10); AFM images and height profile analysis (Figure S11); FTIR spectra (Figure S12); GIWAXD patterns with assigned Miller indices and reciprocal nets (Figure S13); and a schematic illustration of face-on and edge-on irregular nanosheets in a spin-coated film (Figure S14) (PDF)

## References

[1] Discher D. E., Eisenberg A. (2002). Polymer Vesicles. Science.

[2] Li C., Li Q., Kaneti Y. V., Hou D., Yamauchi Y., Mai Y. (2020). Self-Assembly
of Block Copolymers towards Mesoporous Materials for Energy Storage
and Conversion Systems. Chem. Soc. Rev..

[3] Mai Y., Eisenberg A. (2012). Self-Assembly
of Block Copolymers. Chem. Soc. Rev..

[4] Zhang L., Yu K., Eisenberg A. (1996). Ion-Induced
Morphological Changes in “Crew-Cut”
Aggregates of Amphiphilic Block Copolymers. Science.

[5] Gröschel A. H., Walther A., Löbling T. I., Schacher F. H., Schmalz H., Müller A. H. (2013). Guided Hierarchical Co-Assembly of Soft Patchy Nanoparticles. Nature.

[6] Löbling T. I., Borisov O., Haataja J. S., Ikkala O., Gröschel A. H., Müller A. H. (2016). Rational Design of ABC Triblock Terpolymer Solution
Nanostructures with Controlled Patch Morphology. Nat. Commun..

[7] Zhu J., Zhang S., Zhang K., Wang X., Mays J. W., Wooley K. L., Pochan D. J. (2013). Disk-Cylinder
and Disk-Sphere Nanoparticles
Via a Block Copolymer Blend Solution Construction. Nat. Commun..

[8] Pochan D. J., Chen Z., Cui H., Hales K., Qi K., Wooley K. L. (2004). Toroidal Triblock
Copolymer Assemblies. Science.

[9] Cui H., Chen Z., Zhong S., Wooley K. L., Pochan D. J. (2007). Block Copolymer
Assembly Via Kinetic Control. Science.

[10] Hayward R. C., Pochan D. J. (2010). Tailored Assemblies
of Block Copolymers in Solution:
It is All about the Process. Macromolecules.

[11] Qiu H., Hudson Z. M., Winnik M. A., Manners I. (2015). Multidimensional Hierarchical
Self-Assembly of Amphiphilic Cylindrical Block Comicelles. Science.

[12] Qiu H., Gao Y., Boott C. E., Gould O. E., Harniman R. L., Miles M. J., Webb S. E., Winnik M. A., Manners I. (2016). Uniform Patchy and
Hollow Rectangular Platelet Micelles from Crystallizable Polymer Blends. Science.

[13] Deng R., Mao X., Pearce S., Tian J., Zhang Y., Manners I. (2022). Role of Competitive
Crystallization Kinetics in the Formation of 2D Platelets with Distinct
Coronal Surface Patterns via Seeded Growth. J. Am. Chem. Soc..

[14] Teng F., Xiang B., Liu L., Varlas S., Tong Z. (2023). Precise Control
of Two-Dimensional Hexagonal Platelets Via Scalable, One-Pot Assembly
Pathways Using Block Copolymers with Crystalline Side Chains. J. Am. Chem. Soc..

[15] Xia T., Tong Z., Xie Y., Arno M. C., Lei S., Xiao L., Rho J. Y., Ferguson C. T., Manners I., Dove A. P. (2023). Tuning the Functionality of Self-Assembled 2D Platelets
in the Third Dimension. J. Am. Chem. Soc..

[16] Yu X., Fang Y., Luo Z., Guo X., Fu L., Fan Z., Zhao J., Xie H., Guo M., Cheng B. (2025). Precise Preparation
of Size-Uniform Two-Dimensional Platelet Micelles through Crystallization-Assisted
Rapid Microphase Separation Using All-Bottlebrush-Type Block Copolymers
with Crystalline Side Chains. J. Am. Chem. Soc..

[17] Farmer M. A., Musa O. M., Armes S. P. (2024). Combining
Crystallization-Driven
Self-Assembly with Reverse Sequence Polymerization-Induced Self-Assembly
Enables the Efficient Synthesis of Hydrolytically Degradable Anisotropic
Block Copolymer Nano-Objects Directly in Concentrated Aqueous Media. J. Am. Chem. Soc..

[18] Li Z., Zhang Y., Wu L., Yu W., Wilks T. R., Dove A. P., Ding H.-m., O’Reilly R. K., Chen G., Jiang M. (2019). Glyco-Platelets with Controlled Morphologies
via Crystallization-Driven Self-Assembly and their Shape-Dependent
Interplay with Macrophages. ACS Macro Lett..

[19] Liu L., Meng X., Li M., Chu Z., Tong Z. (2024). Regulation
of Two-Dimensional Platelet Micelles with Tunable Core Composition
Distribution via Coassembly Seeded Growth Approach. ACS Macro Lett..

[20] Zhu L., Liu L., Varlas S., Wang R.-Y., O’Reilly R. K., Tong Z. (2023). Understanding the Seeded Heteroepitaxial Growth of Crystallizable
Polymers: The Role of Crystallization Thermodynamics. ACS Nano.

[21] Tang J., Li X. Y., Wu H., Ren L. J., Zhang Y. Q., Yao H. X., Hu M. B., Wang W. (2016). Tube-graft-Sheet Nano-Objects
Created by a Stepwise Self-Assembly of Polymer-Polyoxometalate Hybrids. Langmuir.

[22] Wen T., Qiu L., Zheng Z., Gong Y., Yuan J., Wang Y., Huang M., Yin P. (2020). Inclusion Crystallization
of Silicotungstic
Acid and Poly (Ethylene Oxide) and its Impact on Proton Conductivities. Macromolecules.

[23] Wen T., Zheng Z., Qiu L., Yuan J., Yin P. (2020). Uniform Hybrid
Nanoribbons from Unidirectional Inclusion Crystallization Controlled
by Size-Amphiphilic Block Copolymers. Nanoscale.

[24] Wen T., Wang Y., Yin P., Huang M. (2021). Hybrid Hairy Platelets
with Tunable Structures by Inclusion Crystallization of Polyferrocene-Containing
Block Copolymers and Silicotungstic Acid. ACS
Macro Lett..

[25] Xie S., Sun W., Sun J., Wan X., Zhang J. (2023). Apparent Symmetry Rising
Induced by Crystallization Inhibition in Ternary Co-Crystallization-Driven
Self-Assembly. Nat. Commun..

[26] Akram B., Wang X. (2019). Self-Assembly of Ultrathin
Nanocrystals to Multidimensional Superstructures. Langmuir.

[27] Wang C., Zhao H. (2024). Polymer Brushes and Surface Nanostructures: Molecular Design, Precise
Synthesis, and Self-Assembly. Langmuir.

[28] Topchieva I. N., Tonelli A. E., Panova I. G., Matuchina E. V., Kalashnikov F. A., Gerasimov V. I., Rusa C. C., Rusa M., Hunt M. A. (2004). Two-Phase Channel
Structures Based on α-Cyclodextrin–
Polyethylene Glycol Inclusion Complexes. Langmuir.

[29] Hamley I. W., Castelletto V. (2024). Cyclodextrin-Induced
Suppression of the Crystallization
of Low-Molar-Mass Poly (Ethylene Glycol). ACS
Polym. Au.

[30] Hamley I. W., Castelletto V., Hermida-Merino D., Rosenthal M. (2024). Cyclodextrin-Induced
Suppression of PEG Crystallization from the Melt in a PEG-Peptide
Conjugate. ChemBioChem.

[31] Hwang M. J., Bae H. S., Kim S. J., Jeong B. (2004). Polyrotaxane Hexagonal
Microfiber. Macromolecules.

[32] Chung J. W., Kang T. J., Kwak S.-Y. (2007). Supramolecular
Self-Assembly of Architecturally
Variant α-Cyclodextrin Inclusion Complexes as Building Blocks
of Hexagonally Aligned Microfibrils. Macromolecules.

[33] Schmidt B. V., Hetzer M., Ritter H., Barner-Kowollik C. (2014). Complex Macromolecular
Architecture Design via Cyclodextrin Host/Guest Complexes. Prog. Polym. Sci..

[34] Zhou F., Gu K. H., Zhang Z. Y., Zhang M. Y., Zhou S., Shen Z., Fan X. H. (2016). Exploiting
Host–Guest Interactions
for the Synthesis of a Rod–Rod Block Copolymer with Crystalline
and Liquid-Crystalline Blocks. Angew. Chem.,
Int. Ed..

[35] Chung P., Sun Y.-S., Zhao B.-C., Liu C.-L. (2025). Methylammonium Lead
Halide Nanocubes Templated by Block Copolymer Colloids. Mater. Today Chem..

[36] Chung P., Sun Y.-S., Zhao B.-C., Liu C.-L. (2025). Template-Mediated
Synthesis of Methylammonium Lead Bromide Quantum Nanodots with Tailored
Optical Properties. ACS Appl. Opt. Mater..

[37] Junisu B. A., Sun Y.-S., Septani C. M., Shih O. (2024). CsPbBr_3_ Nanocrystals
Prepared Using Block Copolymer Micelles for LEDs. ACS Appl. Nano Mater..

[38] Sun Y.-S., Wu K.-W., Shih O. (2024). Tuning Perovskite Nanocrystal Synthesis
via Amphiphilic Block Copolymer Templates and Solvent Interactions. ACS Appl. Mater. Interfaces.

[39] Junisu B. A., Sun Y.-S., Zhao B.-C. (2025). Room-Temperature
Synthesis of Highly
Luminescent Methylammonium Lead Bromide Nanocubes Encapsulated in
Block Copolymer Micelles: Impact of Solvent Choice on Crystallization
and Stability. J. Mater. Chem. C.

[40] Yoon S. J., Stamplecoskie K. G., Kamat P. V. (2016). How Lead Halide Complex Chemistry
Dictates the Composition of Mixed Halide Perovskites. J. Phys. Chem. Lett..

[41] Balakrishnan S. K., Kamat P. V. (2018). Ligand Assisted Transformation of
Cubic CsPbBr_3_ Nanocrystals into Two-Dimensional CsPb_2_Br_5_ Nanosheets. Chem. Mater..

[42] Junisu B. A., Chang I. C.-Y., Lin C.-C., Sun Y.-S. (2022). Surface Wrinkling
on Polymer Films. Langmuir.

[43] Beaucage G. (1996). Small-Angle
Scattering from Polymeric Mass Fractals of Arbitrary Mass-Fractal
Dimension. J. Appl. Crystallogr..

[44] Beaucage G. (1995). Approximations
Leading to a Unified Exponential/Power-Law Approach to Small-Angle
Scattering. J. Appl. Crystallogr..

[45] Sun Y.-S., Hu W.-Y., Chung P., Wu K.-W., Shih O., Chuang W.-T. (2024). Poly (Ethylene Oxide)
Crystallization and Gelation
in Butanol Studied by in Situ SAXS/WAXD. Macromolecules.

[46] Zhu L., Cheng S. Z., Calhoun B. H., Ge Q., Quirk R. P., Thomas E. L., Hsiao B. S., Yeh F., Lotz B. (2000). Crystallization
Temperature-Dependent Crystal Orientations within Nanoscale Confined
Lamellae of a Self-Assembled Crystalline– Amorphous Diblock
Copolymer. J. Am. Chem. Soc..

[47] Huq R., Chiodelli G., Ferloni P., Magistris A., Farrington G. (1987). Poly (ethylene
oxide) Complexes of Lead Halides: New
Polymeric Conductors of Pb^2+^. J.
Electrochem. Soc..

[48] Wendsjö Å., Thomas J., Jones G., Farrington G. (1990). Crystallinity
and Thermomechanical Properties of Lead Halide-PEO Complexes. Solid State Ionics.

[49] Fang Y., Giesecke M., Furo I. (2017). Complexing Cations
by Poly (ethylene
oxide): Binding Site and Binding Mode. J. Phys.
Chem. B.

[50] Davidovich R. L., Stavila V., Marinin D. V., Voit E. I., Whitmire K. H. (2009). Stereochemistry
of Lead (Il) Complexes with Oxygen Donor Ligands. Coord. Chem. Rev..

[51] Shimoni-Livny L., Glusker J. P., Bock C. W. (1998). Lone Pair Functionality
in Divalent
Lead Compounds. Inorg. Chem..

[52] Mok M. M., Thiagarajan R., Flores M., Morse D. C., Lodge T. P. (2012). Apparent
Critical Micelle Concentrations in Block Copolymer/Ionic Liquid Solutions:
Remarkably Weak Dependence on Solvophobic Block Molecular Weight. Macromolecules.

[53] van
Stam J., Creutz S., De Schryver F. C., Jérôme R. (2000). Tuning of
the Exchange Dynamics of Unimers Between Block Copolymer Micelles
with Temperature, Cosolvents, and Cosurfactants. Macromolecules.

[54] Young W.-S., Epps III T. H. (2009). Salt Doping in PEO-containing Block Copolymers: Counterion
and Concentration Effects. Macromolecules.

[55] Young W.-S., Albert J. N., Schantz A. B., Epps III T. H. (2011). Mixed-Salt Effects
on the Ionic Conductivity of Lithium-Doped PEO-Containing Block Copolymers. Macromolecules.

[56] Teran A. A., Balsara N. P. (2014). Thermodynamics of Block Copolymers with and without
Salt. J. Phys. Chem. B.

[57] Nakamura I., Balsara N. P., Wang Z.-G. (2013). First-Order
Disordered-to-Lamellar
Phase Transition in Lithium Salt-Doped Block Copolymers. ACS Macro Lett..

[58] Xu Y., Zhang Q., Lv L., Han W., Wu G., Yang D., Dong A. (2017). Synthesis of Ultrasmall CsPbBr_3_ Nanoclusters and their Transformation to Highly Deep-Blue-Emitting
Nanoribbons at Room Temperature. Nanoscale.

[59] Zhang B., Altamura D., Caliandro R., Giannini C., Peng L., De Trizio L., Manna L. (2022). Stable CsPbBr_3_ Nanoclusters
Feature a Disk-Like Shape and a Distorted Orthorhombic Structure. J. Am. Chem. Soc..

[60] Nevers D. R., Williamson C. B., Savitzky B. H., Hadar I., Banin U., Kourkoutis L. F., Hanrath T., Robinson R. D. (2018). Mesophase Formation
Stabilizes High-Purity Magic-Sized Clusters. J. Am. Chem. Soc..

[61] Young W.-S., Brigandi P. J., Epps T. H. (2008). Crystallization-induced lamellar-to-lamellar
thermal transition in salt-containing block copolymer electrolytes. Macromolecules.

[62] Hsiao Y.-J., Huang Z.-E., Sahare A., Chen M.-Z., Lin Y.-H., Chen H.-L. (2024). Accessing the Frank–Kasper
Phase of Block Copolymer
via Selective Incorporation of Metal Salt. Macromolecules.

[63] Chintapalli M., Le T. N., Venkatesan N. R., Mackay N. G., Rojas A. A., Thelen J. L., Chen X. C., Devaux D., Balsara N. P. (2016). Structure
and Ionic Conductivity of Polystyrene-block-Poly (ethylene oxide)
Electrolytes in the High Salt Concentration Limit. Macromolecules.

[64] Cai R., Yang D., Lin K.-T., Lyu Y., Zhu B., He Z., Zhang L., Kitamura Y., Qiu L., Chen X. (2019). Generalized Preparation of Two-Dimensional Quasi-Nanosheets via Self-Assembly
of Nanoparticles. J. Am. Chem. Soc..

[65] Xu F., Zhang P., Zhang J., Yu C., Yan D., Mai Y. (2018). Crystallization-Driven Two-Dimensional
Self-Assembly of Amphiphilic
PCL-b-PEO Coated Gold Nanoparticles in Aqueous Solution. ACS Macro Lett..

[66] Chen W. Y., Zheng J. X., Cheng S. Z., Li C. Y., Huang P., Zhu L., Xiong H., Ge Q., Guo Y., Quirk R. P., Lotz B., Deng L., Wu C., Thomas E. L. (2004). Onset of
Tethered Chain Overcrowding. Phys. Rev. Lett..

[67] Mei S., Qi H., Zhou T., Li C. Y. (2017). Precisely Assembled Cyclic Gold Nanoparticle
Frames by 2D Polymer Single-Crystal Templating. Angew. Chem., Int. Ed..

[68] Sun Y. S., Zhao B. C., Shih O., Chen C. Y., Su C. J., Lin J. M. (2025). Tunable Formation of Two-Dimensional Nanostructures
and Complex Crystals via Lead­(II) Bromide Complexation and Inclusion
Crystallization with Polystyrene-Block-Poly­(ethylene oxide) in 1,3,5-Trimethylbenzene. Nanoscale.

