# Supplementary material for: Hybrid Hairy Two-Dimensional Nanostructures with Tunable Morphologies by Inclusion Crystallization of Lead Bromide Complexes with Polystyrene-block-poly(ethylene oxide)
Source: Langmuir. 2025 Nov 18;41(47):31780–91. doi: 10.1021/acs.langmuir.5c03419 (PMC12676742; doi:10.1021/acs.langmuir.5c03419)
Supplement: Supplementary file 1 [file la5c03419_si_001.pdf]

Supporting Information

Hybrid Hairy Two-dimensional Nanostructures with Tunable Morphologies by Inclusion Crystallization of Lead Bromide Complexes with Polystyrene-*block*-poly(ethylene oxide).

Ya-Sen Sun[1*], Bo-Cheng Zhao[1], Chun-Chuen Yang[2], Orion Shih[3], Chun-Yu Chen[3], Chun-Jen Su[3], and Jhih-Min Lin[3]

1. *Department of Chemical Engineering, National Cheng Kung University, Tainan 701, Taiwan*
2. *Department of Physics, National Central University, Taoyuan City 320317, Taiwan.*
3. *National Synchrotron Radiation Research Center, Hsinchu 30076, Taiwan*

*corresponding author: Y. S. Sun (Email: yssun@gs.ncku.edu.tw)

Number of pages: 21
Number of figures: 14
Number of tables: 2

A table of contents

Figure S1 Photographs and UV–vis absorbance spectra of control solutions P.S2
Table S1 Structural parameters used for SAXS fitting P.S3
Figure S2 SAXS and WAXD profiles of a BCP_{20} solution P.S4
Figure S3 In house WAXD profiles of p-$PRE_{m/n}$ precipitates P.S5
Table S2 Miller indices, q values, d-spacings and q-ratios P.S6-S7
Figure S4 Bright-field TEM images and ED patterns of worm-like nanodomains formed in c-$PRE_{m/n}$ solutions P.S8
Figure S5 Bright-field TEM images and EDS profiles of worm-like nanodomains P.S9
Figure S6 Bright-field TEM image collected for a t-$PRE_{20/10}$ solution P.S9
Figure S7 Bright-field TEM images of a BCP_{20} solution P.S9
Figure S8 Low- and high-magnification bright-field TEM images of dried samples prepared by prolonged stirring P.S11
Figure S9 Bright-field TEM images of c-$PRE_{m/n}$ solutions P.S12
Figure S10 Bright-filed and high-angle annular dark-field images of irregular nanosheets and polygonal nanoplates P.S14
Figure S11 AFM images and height profile analysis P.S16
Figure S12 FTIR spectra P.S18
Figure S13 GIWAXD patterns with assigned Miller indices and reciprocal nets P.S19
Figure S14 Schematic illustration of face-on and edge-on irregular nanosheets in a spin-coated film P.S20
References P.S21

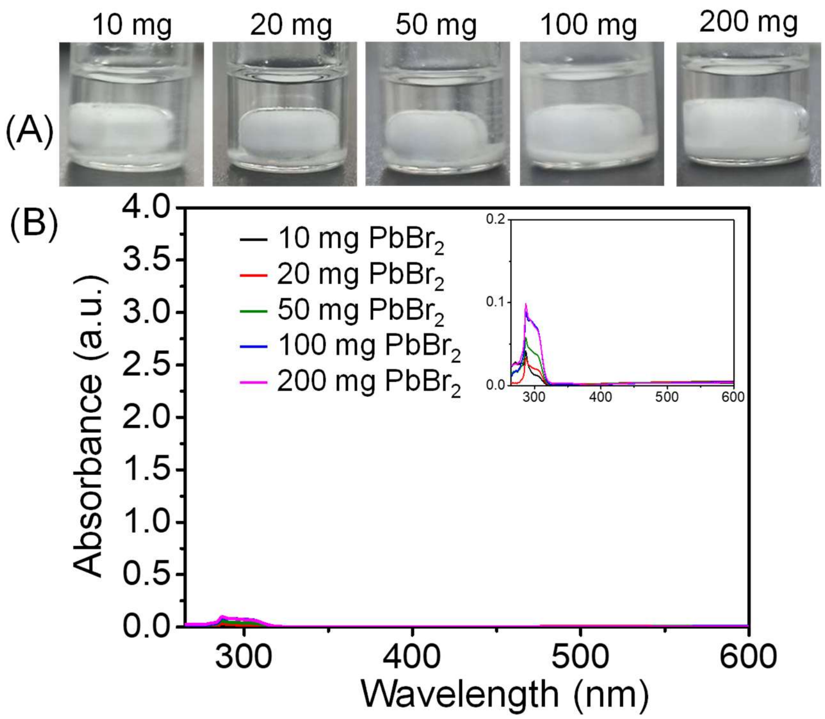

Figure S1. (A) Photographs and (B) UV–vis absorbance spectra of control solutions containing only $PbBr_2$ of different amounts in TMB, without PS-b-PEO. The spectra in (B) were recorded directly, without dilution, after 24 h of stirring. Insert in (B) is an enlarged profile for visualization.

To evaluate the solubility of $PbBr_2$ in TMB without PS-b-PEO, we conducted a control experiment by attempting to dissolve $PbBr_2$ (10, 20, 50, 100, and 200 mg) in neat TMB (1 mL) under stirring at 800 rpm for 24 h. The resulting mixtures were analyzed by UV–vis absorbance spectroscopy, which is highly sensitive to $[Pb_xBr_y]^{2x-y}$ complexes even at very low concentrations. As shown in Figure S1, all samples exhibited visible precipitation of $PbBr_2$, and no absorption bands attributable to $[Pb_xBr_y]^{2x-y}$ complexes were detected in the spectra (Figure S1B). These results confirm that $PbBr_2$ is essentially insoluble in neat TMB.

Table S1: A summary of structural parameters used for fitting Figure 1C.

[a] radius of gyration, exponent	c-$PRE_{20/10}$	c-$PRE_{20/100}$	c-$PRE_{20/200}$
2-level Beaucage model			
$R_{g,S}$/Å	79.0	70.6	68.7
α_S	2.9	2.8	2.9
$R_{g,L}$/Å	679.4	623.3	560.8
α_L	3.5	3.2	3.2

a: the subscripts, S, and L represent small and large structures, respectively.

The extracted structural parameters include $R_{g,S}$ and α_S for small structures, and $R_{g,L}$ and α_L for large structures, where R_g represents the radius of gyration and α denotes the Porod exponent (Table S1, Supporting Information). Table S1 reveals that the small structures exhibit $R_{g,S}$ values of a few nanometers, with α_s ~2.8. In contrast, the large structures have $R_{g,L}$ values in the tens of nanometers, with α_L ranging from 3.2 to 3.5. As compared with the morphological observations from TEM (Figures S8–S10), the small structures ($R_{g,S}$ of a few nanometers, α_S ~2.8–2.9) likely correspond to tiny $PbBr_2$ nanoparticles, whereas the larger structures ($R_{g,L}$ in the tens of nanometers, α_L ~3.2–3.5) are attributed to 2D nanostructures with rough surfaces.

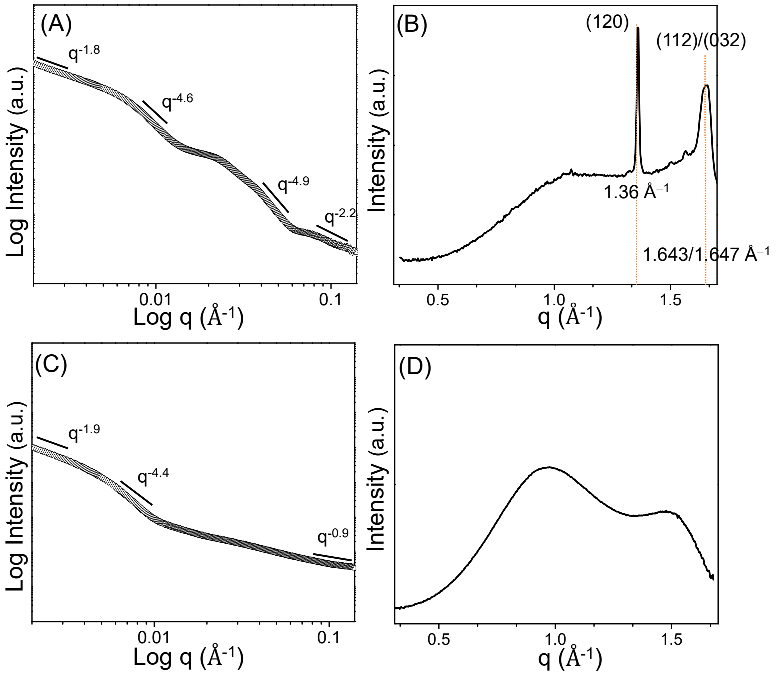

Figure S2. (A,C) SAXS and (B, D) WAXD profiles of a BCP_{20} solution that contains 20 mg PS-b-PEO in 1mL TMB. The profiles were measured at (A,B) 25 ºC or (C,D) 80 ºC, respectively.

We performed SAXS and WAXD measurements on a control BCP_{20} solution (20 mg mL^{-1} PS-b-PEO in TMB) both at 25 and 80 °C. The results are shown in Figure S2. we prepared a control BCP_{20} solution (20 mg mL^{-1} PS-b-PEO in TMB) and performed SAXS and WAXD measurements at 25 ˚C and 80 ˚C (Figure S2) to examine how structural evolution influences scattering features. At 25 °C, the SAXS profile (Figure S2A) exhibits multi-level Guinier/Porod-like features similar to those described in the Beaucage unified model[S1], consistent with the coexistence of multiple structures. One of these structures originates from crystallization-driven self-assembly, as confirmed by the WAXD diffraction peaks at q = 1.36, 1.643, and 1.647 $Å^{-1}$ corresponding to monoclinic PEO crystals (Figure S2B). Upon heating to 80 °C, where PEO crystallites are completely melted (Figure S2D), the SAXS profile (Figure S2C) changes markedly: the Guinier/Porod-like features in the q range of 0.01–0.14 $Å^{-1}$ disappear. This comparison indicates that the Guinier/Porod-like features in Figure S2A originate from

PS-b-PEO crystalline microplates. Notably, the low-q regions ($q < 0.007$ Å^{-1}) of Figures S2A and S2C both show an intensity decay of $I \sim q^{-1.8}$–$q^{-1.9}$, deviating from the $I \sim q^{-1}$ behavior expected for rigid cylinders[S2]. Instead, this slope is more consistent with nanobelts and nanoribbons,[S3-S5] suggesting that PS-b-PEO in TMB at 80 °C tends to form belt-like nanostructures rather than rod-like nanostructures.

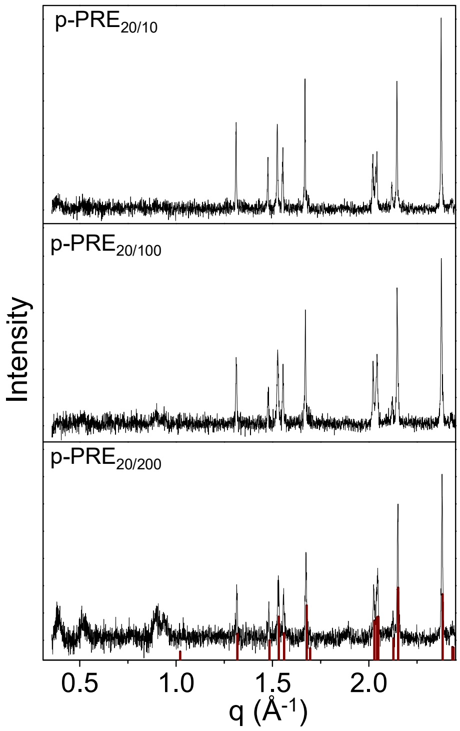

Figure S3. In house WAXD profiles of p-PRE$_{m/n}$ precipitates with m/n=20/10, 20/100 and 20/200. The p-PRE$_{m/n}$ precipitates were separated from the c-PRE$_{m/n}$ solutions by centrifuging the t-PRE$_{m/n}$ solutions. The stick pattern represents the orthorhombic $PbBr_2$ crystal (PDF #031-0679)

Different from the WAXD profiles of the supernatants, the in-house WAXD profile of p-PRE$_{m/n}$ precipitates that were separated from the c-PRE$_{m/n}$ supernatants show diffraction peaks of $PbBr_2$ orthorhombic crystals with Pnma symmetry (Figure S3). This comparison indicates that the centrifugation process predominately removed $PbBr_2$ microparticles.

Table S2. Miller indices, q, d-spacing and q-ratio values estimated for the additional diffraction peaks shown in Figure 1D.

c-PRE$_{20/10}$					
orthorhombic complex crystal with Cmca symmetry (a=23.53 Å, a=4.20 Å, c=33.22 Å; α=β=γ=90°)					
[a]q-obs/Å^{-1}	h	k	l	[b]q-calc/Å^{-1}	[c]d-calc/Å
0.388	0	0	2	0.378	16.61
0.539	2	0	0	0.534	11.76
0.949	2	0	4	0.945	6.65
1.073	4	0	0	1.068	5.88
1.565	1	1	2	1.568	4.00
1.748	3	1	2	1.742	3.61
1.870	3	1	4	1.867	3.36
1.917	1	1	6	1.918	3.28
1.956	3	1	5	1.957	3.21
2.039	5	1	2	2.043	3.08
2.094	5	1	3	2.089	3.01
2.263	8	0	4	2.274	2.76
2.404	7	1	1	2.402	2.62
2.439	3	1	9	2.441	2.67
hexagonal complex crystals with P6mm symmetry (a=b=13.954 Å, γ=120°)					
q-obs/Å^{-1}	h	k	[d]q ratio	q-calc/Å^{-1}	d-calc/Å
0.522	1	0	1	0.522	12.04
0.902	1	1	$3^{1/2}$	0.903	6.96
1.043	2	0	2	1.069	5.88
1.375	2	1	$7^{1/2}$	1.373	4.58
1.564	3	0	3	1.605	3.91
1.802	2	2	$12^{1/2}$	1.801	3.49
1.896	3	1	$13^{1/2}$	1.875	3.35
2.099	4	0	4	2.088	3.02
2.262	3	2	$19^{1/2}$	2.275	2.77
2.387	4	1	$21^{1/2}$	2.392	2.64

c-PRE$_{20/100}$ 、c-PRE$_{20/200}$					
orthorhombic complex crystal with Cmca symmetry (a=23.53 Å, a=4.20 Å, c=33.22 Å; α=β=γ=90°)					
[a]q-obs/Å^{-1}	h	k	l	[b]q-calc/Å^{-1}	[c]d-calc/Å
0.386	0	0	2	0.378	16.61
0.539	2	0	0	0.534	11.76
0.659	2	0	2	0.654	9.60
0.780	0	0	4	0.780	8.06
0.944	2	0	4	0.945	6.65
1.073	4	0	0	1.068	5.88
1.142	4	0	2	1.137	5.53
1.267	2	0	6	1.286	4.89
1.319	4	0	4	1.323	4.75
1.564	0	0	8	1.560	4.03
1.568	1	1	2	1.569	4.00
1.586	4	0	6	1.584	3.97
1.607	6	0	0	1.602	3.92
1.646	6	0	2	1.649	3.81
1.745	3	1	2	1.741	3.61
1.776	6	0	4	1.782	3.53
1.797	3	1	3	1.795	3.50
1.866	3	1	4	1.868	3.36
1.896	4	0	8	1.891	3.32
1.917	1	1	6	1.918	3.28
1.960	3	1	5	1.957	3.21
1.995	5	1	1	2.013	3.12
2.025	2	0	10	2.022	3.11
2.038	5	1	2	2.043	3.08
2.090	5	1	3	2.089	3.01
2.141	8	0	0	2.136	2.94
2.172	3	1	7	2.178	2.89
2.279	8	0	4	2.274	2.76
2.404	7	1	1	2.402	2.62
2.443	3	1	9	2.441	2.57
hexagonal complex crystals with P6mm symmetry (a=b=13.954 Å, γ=120°)					
q-obs/Å^{-1}	h	k	[d]q ratio	q-calc/Å^{-1}	d-calc/Å
0.522	1	0	1	0.522	12.04
0.902	1	1	$3^{1/2}$	0.903	6.96
1.043	2	0	2	1.069	5.88
1.375	2	1	$7^{1/2}$	1.373	4.58
1.564	3	0	3	1.605	3.91
1.802	2	2	$12^{1/2}$	1.801	3.49
1.896	3	1	$13^{1/2}$	1.875	3.35
2.099	4	0	4	2.088	3.02
2.262	3	2	$19^{1/2}$	2.275	2.77
2.387	4	1	$21^{1/2}$	2.392	2.64

Superscripts: ***a***. q-obs: observed q values; ***b***. q-calc: calculated q values; ***c***. d-calc: calculated d-spacing values; ***d***. q ratio: the ratio of the high-order diffraction peaks with respect to the first-order peak.

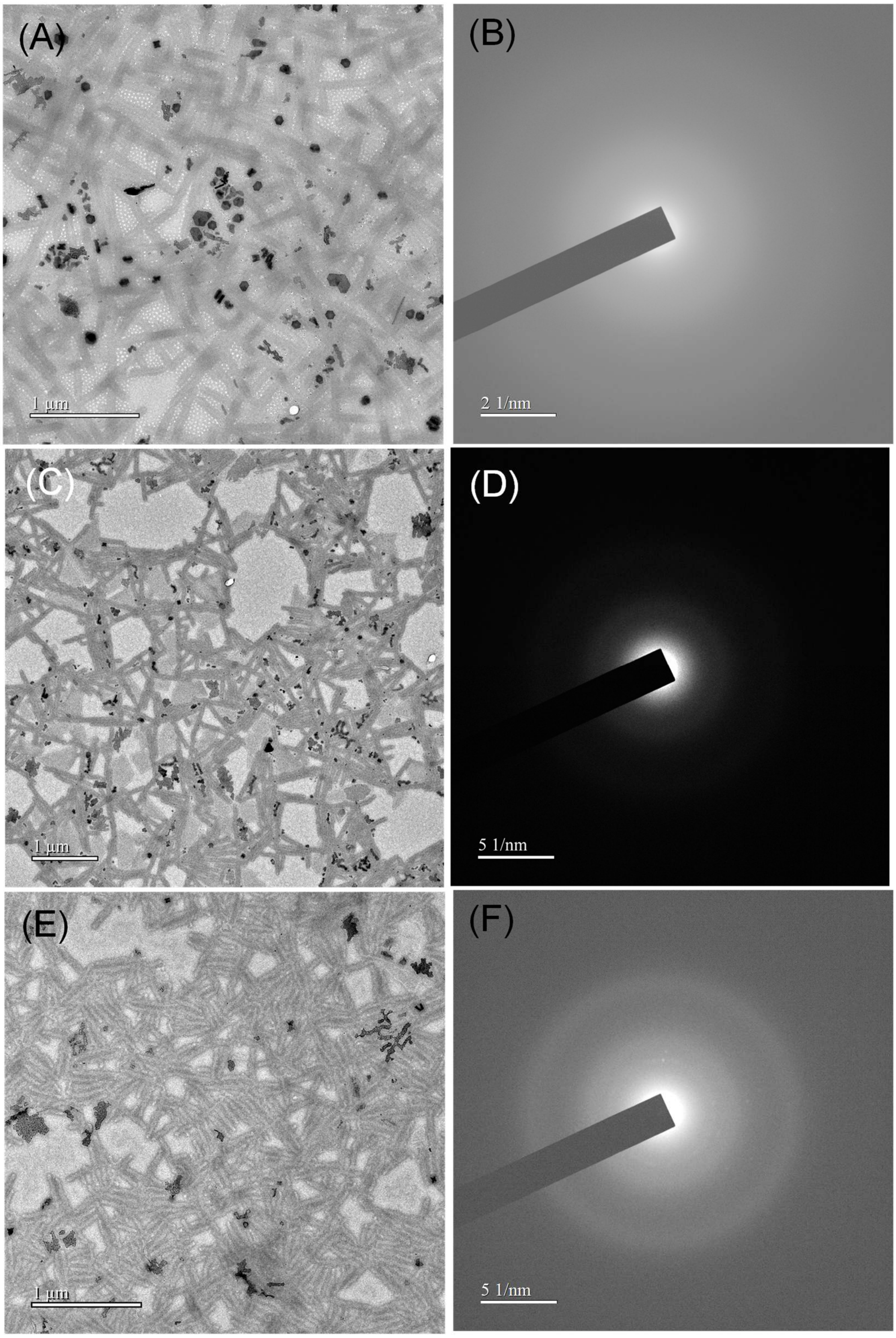

Figure S4. (A, C, E) Bright-field TEM images and (B, D, F) corresponding ED patterns of worm-like nanodomains observed in dried samples from (A, B) c-$PER_{20/10}$ (C, D) c-$PRE_{20/100}$, and (E, F) c-$PER_{20/200}$ solutions.

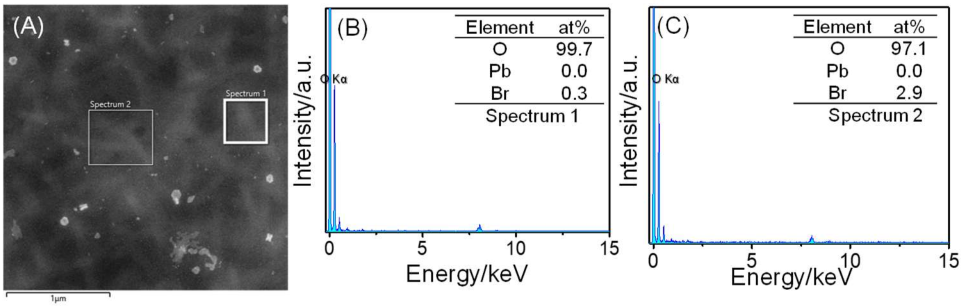

Figure S5. (A) High-angle annular dark-field TEM image and (B, C) corresponding EDS profiles collected from a dried c-$PRE_{20/10}$ solution. In (A), the positions selected for EDS analysis are indicated by white boxes, which contain only worm-like nanodomains.

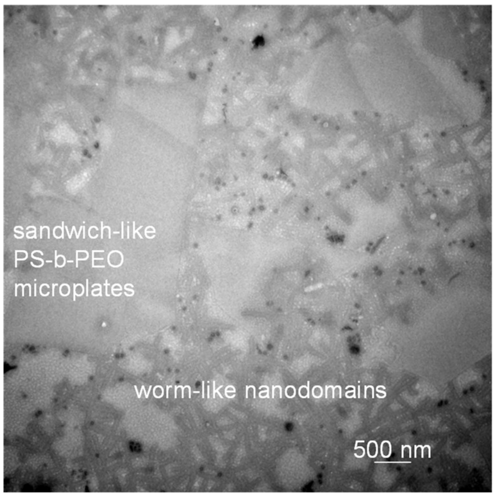

Figure S6. Bright-field TEM image showing sandwich-like microplates coexisting with worm-like nanodomains and hairy 2D nanostructures in the dried state of a t-$PRE_{20/10}$ solution.

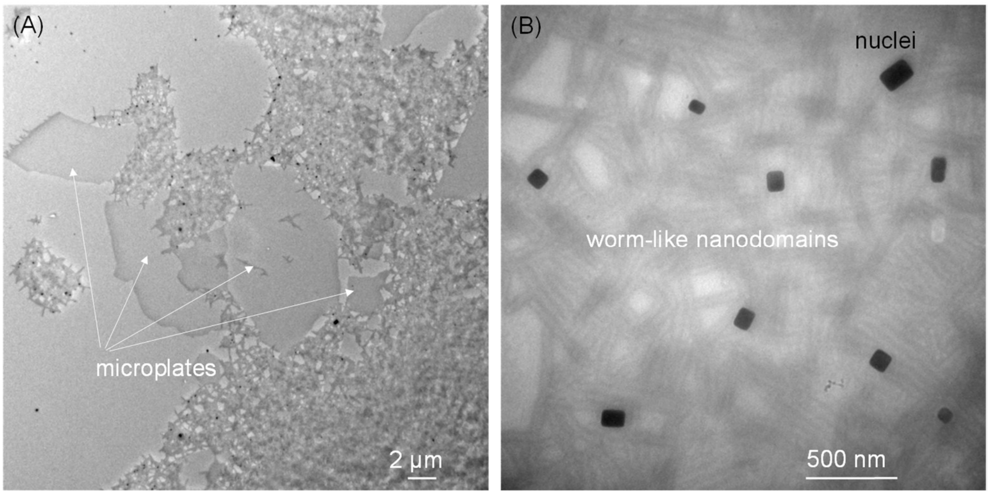

Figure S7. (A) low-magnification and (B) large-magnification bright-field TEM images of the dried state of a BCP_{20} solution.

Figure S4 shows low-voltage bright-field TEM images and SAED patterns collected from the dried precursor supernatants. As seen in Figures S4A/C/E, the dried films are filled with worm-like nanodomains coexisting with hairy two-dimensional nanostructures. ED and EDS characterization confirm that the worm-like nanodomains are amorphous and primarily composed of PS-b-PEO (Figures S4B/D/F and S5). In addition, sandwich-like microplates are also observed to coexist with worm-like nanodomains and hairy 2D nanostructures (Figure S6). Notably, even before the addition of $PbBr_2$, both worm-like nanodomains and sandwich-like microplates were already present in BCP_{20} solutions (Figure S7). It is well established that crystallization of the PEO block in PS-b-PEO produces sandwich-like microplates, each consisting of a compact PEO crystalline lamella sandwiched between two layers of PS brushes[S6–S8]. Based on this, we infer that the worm-like nanodomains arise from incompatibility-driven self-assembly of PS-b-PEO chains without involving $PbBr_2$-based complexes, while the microplates are generated through crystallization-driven self-assembly of PS-b-PEO chains.

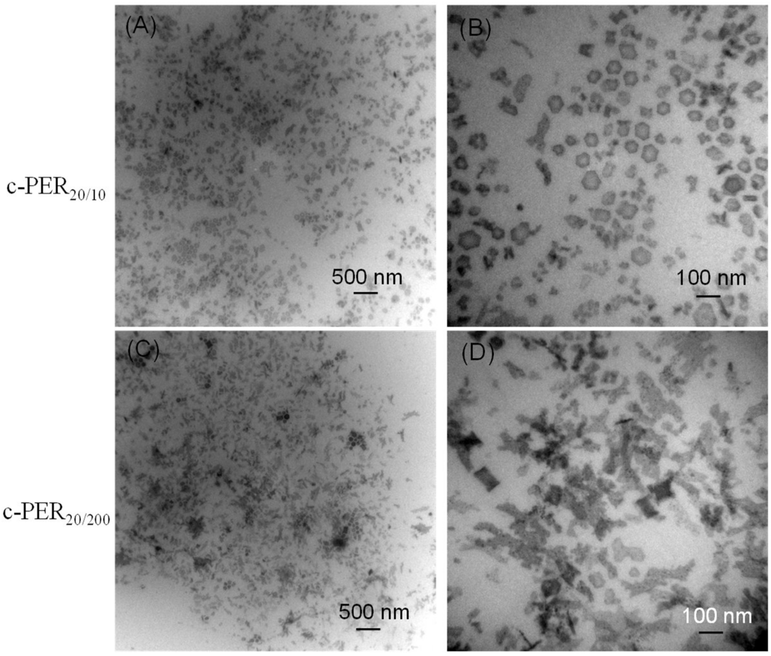

Figure S8. Low- and high-magnification bright-field TEM images of dried samples from (A, B) c-$PRE_{20/10}$, and (C, D) c-$PRE_{20/200}$ solutions that were prepared by prolonged stirring (3 days) at 800 rpm and then centrifugated at 7000 rpm (3 min).

To understand if the 2D nanostructures form thermodynamically or kinetically, we prepared 2D nanostructures at the weight ratios of PS-b-PEO/$PbBr_2$=20/10 and 20/200 by prolonged stirring PS-b-PEO/$PbBr_2$ hybrids (three days) at 800 rpm in TMB. After 3d stirring, the solutions were centrifugated to remove $PbBr_2$ microparticles and then drop casted as films for TEM characterization. The trend shown in Figure S8 is consistent with the trend discussed for Figure 2. This is, polygonal nanoplates are favored to grow at 20/10 while irregular nanosheets are favored to grow at 20/200. This comparison indicates that these structures are thermodynamically stable, rather than kinetically trapped.

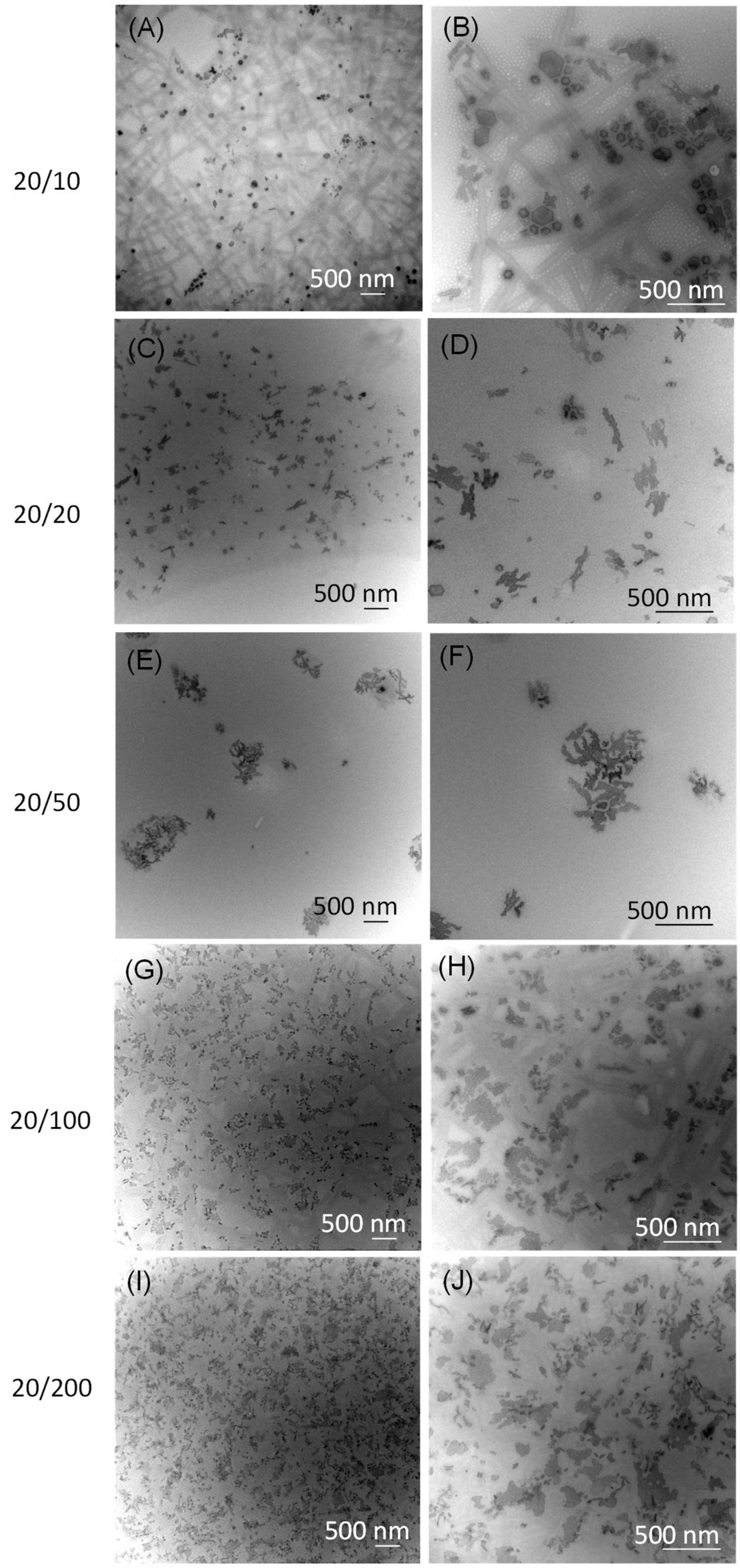

Figure S9. Low- and high-magnification bright-field TEM images of dried samples from (A, B) c-$PRE_{20/10}$, (C, D) c-$PRE_{20/20}$, (E, F) c-$PRE_{20/50}$ (G, H) c-$PRE_{20/100}$, and (I, J) c-$PRE_{20/200}$ solutions.

As seen in Figure S9, polygonal nanoplates are favored at the 20/10 condition, while progressively increasing the $PbBr_2$ content shifts the morphology toward irregular nanosheets. Notably, at ratios of 20/50, 20/100, and 20/200, irregular nanosheets become the major morphology, indicating that beyond a certain $PbBr_2$ concentration, they dominate over polygonal nanoplates. These additional results further support our conclusion that the relative concentrations of PS-b-PEO and $PbBr_2$ critically govern the balance between polygonal nanoplates and irregular nanosheets.

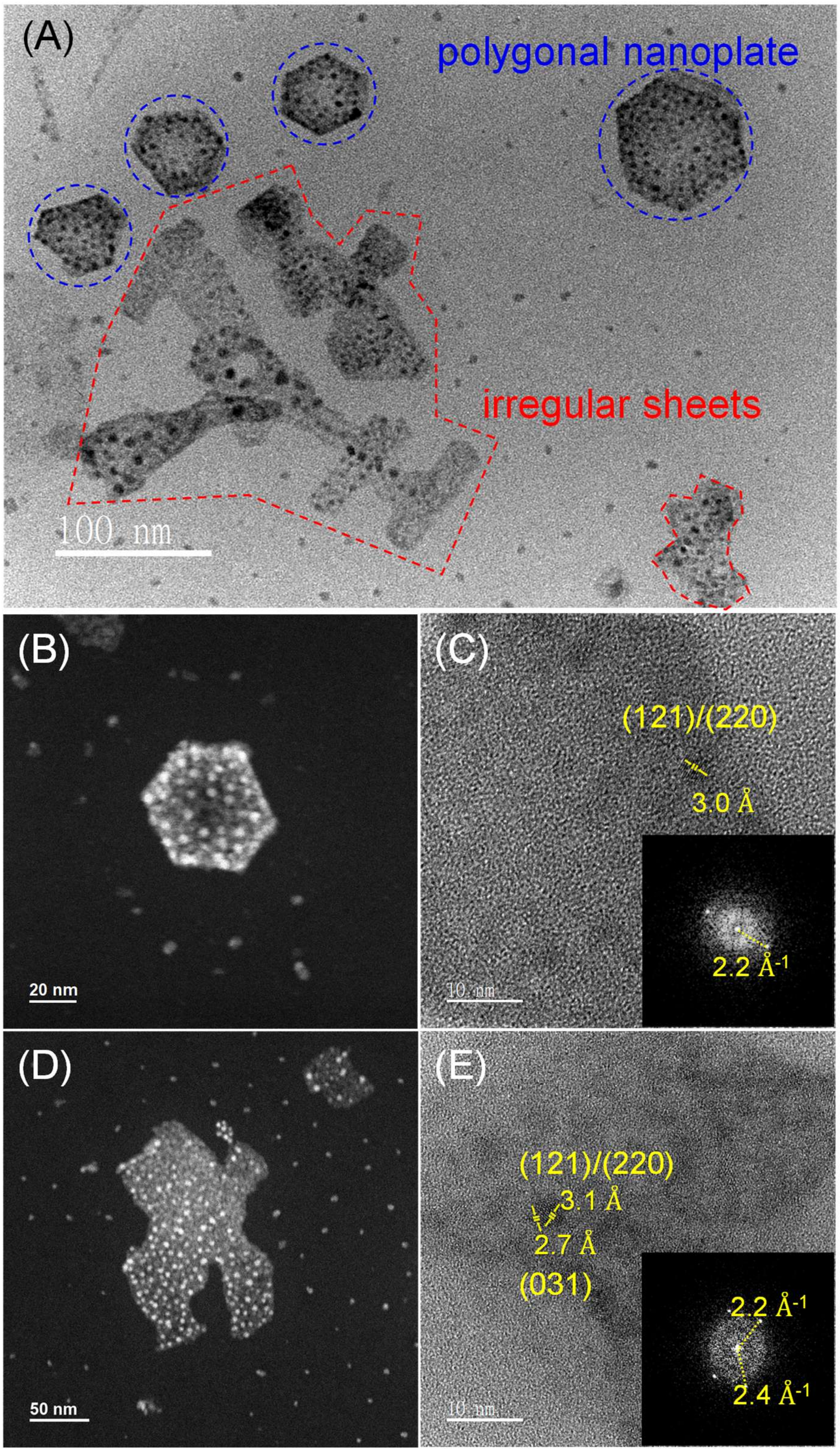

Figure S10. (A) low-magnification bright-field TEM image showing a mixture of irregular nanosheets (marked in red) and polygonal nanoplates (marked in blue). (B, D) high-angle annular dark-field, and (C, E) bright-field TEM images of selected (B, C) polygonal nanoplate and (D, E) irregular nanosheet. Insets in (C, E) display FFT patterns of selected nanodots exhibiting lattice fringes. All data were collected from the c-PRE$_{20/10}$ solution in a dried state.

Figure S10 show ultra-high resolution bright-field TEM images collected on aggregates of irregular nanosheets and polygonal nanoplates. The irregular nanosheet and polygonal nanoplate were covered by numerous tiny nanodots. The tiny nanodots are comprised of $PbBr_2$ orthorhombic crystals, evidenced by (121)/(220) and (031) lattice fringes in the images and diffraction spots in the FFT patterns (Figure S10C/E). Additionally, we observe several free tiny nanodots near 2D nanostructures. The tiny nanodots do not cover the 2D nanostructures' surface. In contrast, sandwich-like microplates are free from the decoration of tiny $PbBr_2$ nanoparticles onto their surfaces (Figure S6).

TEM lattice imaging is a valuable technique for inorganic materials with strong metallic or ionic bonding. However, due to the polymer content and the relatively weak, molecular-level interactions between PEO segments and the $[Pb_xBr_y]^{2x-y}$ complexes, lattice fringes or moiré patterns cannot be readily observed under conventional TEM conditions. Observation of lattice fringes typically requires ultrahigh-resolution TEM with strong electron irradiation, which is not feasible here because polymer-containing hybrid crystals are highly sensitive to radiation damage. Under such conditions, prolonged exposure leads to rapid degradation of both the polymer matrix and the coordination complexes, obscuring any lattice textures. Furthermore, the surface decoration of BCP-capped tiny nanoparticles onto the 2D complex crystals also disturb surface observation of lattice fringes in TEM.

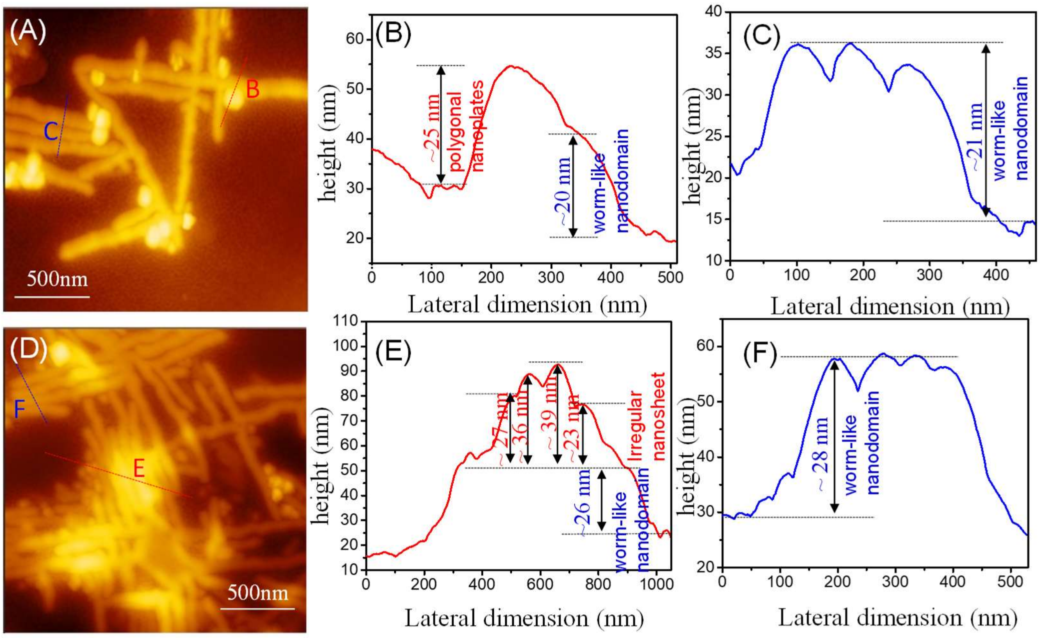

Figure S11. (A, D) AFM topographic images and (B-C and E-F) height profiles measured for thin films obtained by spin coating (3000 rpm, 1 min) (A-C) c-$PRE_{20/10}$ and (D-F) c-$PRE_{20/200}$ solutions.

To determine the thicknesses of these structures, AFM measurements were carried out. Because AFM provides only local information, three different regions of each sample were analyzed to minimize sampling bias. Representative AFM images and height profiles are shown in Figure S11. As evident in Figure S11, the 2D nanostructures inevitably coexist with PS-b-PEO worm-like nanodomains, consistent with the morphological observations in Figure S9. In TEM (Figure S9), irregular nanosheets and polygonal nanoplates are readily identified due to their high contrast, whereas PS-b-PEO worm-like nanodomains appear with much lower contrast. In contrast, AFM does not provide such strong contrast, and the 2D nanostructures have surfaces enriched with tiny $PbBr_2$ nanoparticles. These two factors make precise height determination challenging.

Nevertheless, careful inspection based on the AFM characterization reveals that polygonal nanoplates preferentially lie adjacent to PS-b-PEO worm-like nanodomains

(Figure S11A), while irregular nanosheets more often overlie them (Figure S11D). The corresponding height profiles indicate average thicknesses of ~25 nm for polygonal nanoplates (Figure S11B) and ~31 nm for irregular nanosheets (Figure S11E). These values should be regarded as approximate, as surface decoration by $PbBr_2$ nanoparticles can locally increase the measured thickness. For comparison, worm-like nanodomains exhibit average thicknesses of ~21 nm in the dried c-$PRE_{20/10}$ sample and ~28 nm in the dried c-$PRE_{20/200}$ sample (Figures S11C/F).

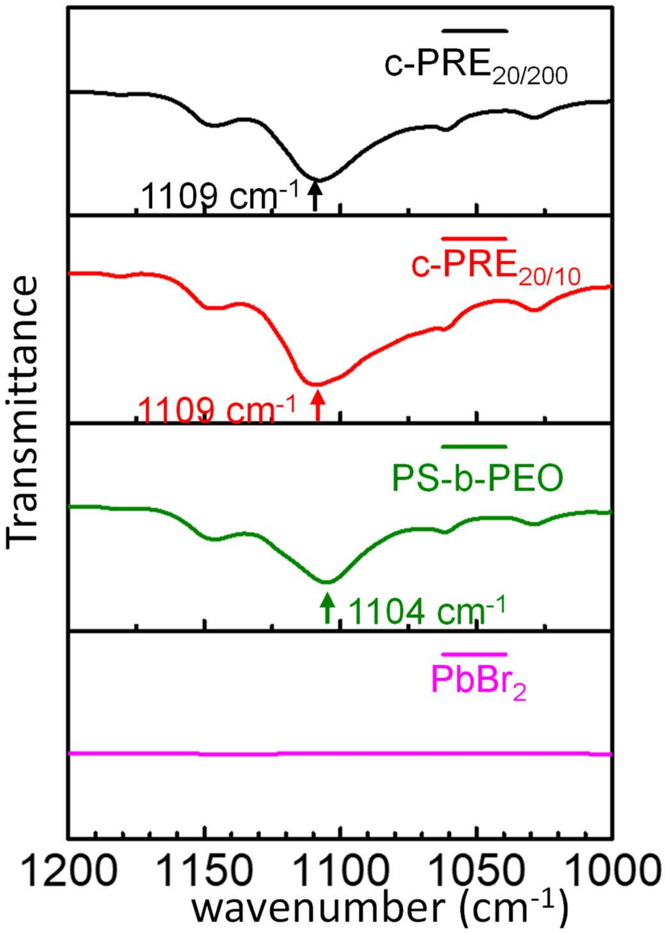

Figure S12. FTIR spectra of hybrids prepared by drop-casting c-$PRE_{20/10}$ and c-$PRE_{20/200}$ solutions, shown alongside the spectra of neat PS-b-PEO and $PbBr_2$ powders for comparison.

To examine the interaction between PEO ether groups and Pb^{2+} cations, we performed FTIR analyses on specimens prepared by drop casting c-$PRE_{20/10}$ and c-$PRE_{20/200}$ solutions. For comparison, neat PS-b-PEO and $PbBr_2$ powders were also characterized. In the FTIR spectra (Figure S12), we focused on the 1000–1200 cm^{-1} region, which corresponds to the C–O stretching vibrations of PEO[S9]. Neat $PbBr_2$ shows no distinct absorption bands in this region, whereas the C–O band shifts from ca 1104 cm^{-1} in neat PS-b-PEO to 1109 cm^{-1} in the PS-b-PEO/$PbBr_2$ hybrids, indicating coordination between the ether groups and Pb^{2+} cations.

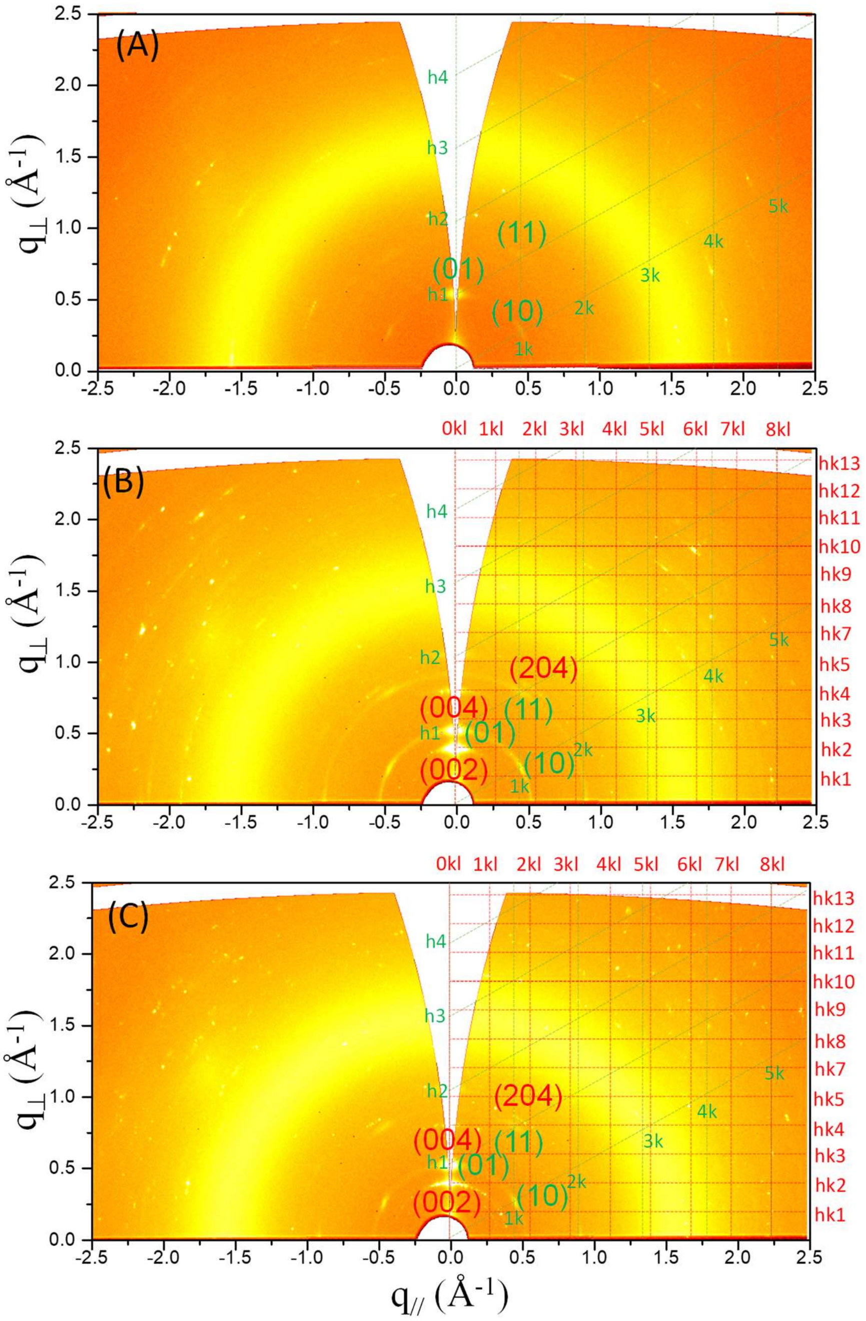

Figure S13. GIWAXD patterns of spin-coated films prepared from c-PRE$_{m/n}$ solutions with m/n = (A) 20/10, (B) 20/100, and (C) 20/200. The green and red reciprocal nets indicate dominant fiber-like orientations of two distinct crystal types: hexagonal complex crystals (green), where the *c*-axis is aligned parallel to the substrate in edge-on polygonal nanoplates, and orthorhombic complex crystals (red), where the *c*-axis is perpendicular to the substrate in face-on irregular nanosheets. Both nanostructures exhibit random in-plane orientation within the *x*–*y* plane of the substrate. Diffraction spots labeled in green originate from hexagonal complex crystals with *P6mm* symmetry, while those in red correspond to orthorhombic complex crystals with *Cmca* symmetry.

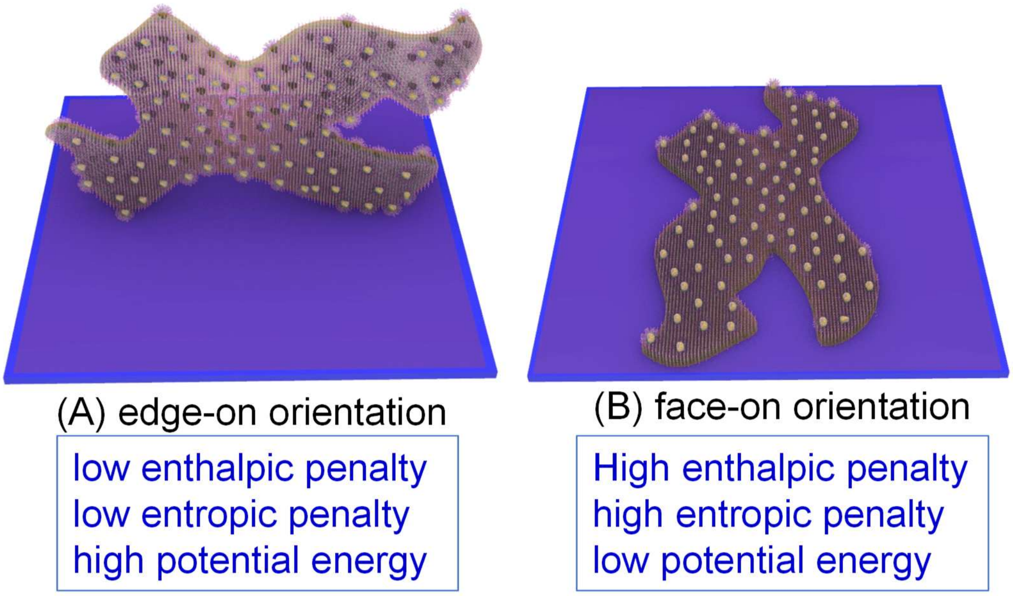

Figure S14. Schematic illustrations of (A) an edge-on and (B) a face-on irregular nanosheet within a spin-coated film. A face-on orientation experiences a significant enthalpic penalty because the PS blocks make unfavorable contacts with the hydrophobic substrate surface. Additionally, compression of the PS chains near the substrate imposes an entropic penalty. In contrast, these penalties are largely absent for the edge-on orientation; however, the large lateral dimensions and irregular edges of the nanosheets may introduce additional potential energy in the edge-on orientation.

References:

S1. Beaucage, G.; Aubert, J. H.; Lagasse, R. R.; Schaefer, D. W; Rieker, T. P.; Erlich, P.; Stein, R. S.; Kulkarni, S.; Whaley, P. D. Nano-structured, Semicrystalline Polymer Foams. *Journal of Polymer Science Part B: Polymer Physics* **1996**, *34,* 3063–3072.

S2. Jiang, N.; Yu, T.; Darvish, O. A.; Qian, S.; Tsengam, I. K. M.; John, V.; Zhang, D. Crystallization-Driven Self-Assembly of Coil–Comb-Shaped Polypeptoid Block Copolymers: Solution Morphology and Self-assembly Pathways. *Macromolecules* **2019**, *52*, 8867–8877.

S3. Cui, H.; Muraoka, T.; Cheetham, A. G.; Stupp, S. I. Self-assembly of Giant Peptide Nanobelts. *Nano Lett.* **2009**, *9*, 945–951.

S4. Dhasaiyan, P.; Prevost, S.; Baccile, N.; Prasad, B. L. V. pH-and Time-Resolved in Situ SAXS Study of Self-Assembled Twisted Ribbons Formed by Elaidic Acid Sophorolipids. *Langmuir* **2018**, *34*, 2121–2131.

S5. Hamley, I. W. Form Factor of Helical Ribbons. *Macromolecules* **2008**, *41*, 8948–8950.

S6. Lotz, B.; Kovacs, A. Propriétés des Copolymères Biséquencés Polyoxyéthylène-Polystyrène: I. Préparation, Composition et étude Microscopique des Monocristaux. *Kolloid-Zeitschrift und Zeitschrift für Polymere* **1966**, *209*, 97-114.

S7. Lotz, B.; Kovacs, A.; Bassett, G.; Keller, A. Properties of Copolymers Composed of One Poly-Ethylene-Oxide and One Polystyrene Block: II. Morphology of Single Crystals. *Kolloid-Zeitschrift und Zeitschrift für Polymere* **1966**, *209*, 115-128.

S8. Chen, W. Y.; Li, C. Y.; Zheng, J. X.; Huang, P.; Zhu, L.; Ge, Q.; Quirk, R. P.; Lotz, B.; Deng, L.; Wu, C.; Thomas, Edwin L.; Cheng, Stephen Z. D. "Chemically Shielded" Poly (Ethylene Oxide) Single Crystal Growth and Construction of Channel-Wire Arrays with Chemical and Geometric Recognitions on a Submicrometer Scale. *Macromolecules* **2004**, *37*, 5292-5299.

S9. Chang Y. H.; Ku, C. W.; Zhang, Y. H.; Wang, H. C.; Chen, J. Y. Ultrafast Responsive Non-Volatile Flash Photomemory via Spatially Addressable Perovskite/Block Copolymer Composite Film. *Adv. Func. Mater*. **2020**, *30*, no 2000764.
